# The Effect of Intramolecular Hydrogen Bond Type on the Gas-Phase Deprotonation of *ortho*-Substituted Benzenesulfonic Acids. A Density Functional Theory Study

**DOI:** 10.3390/molecules25245806

**Published:** 2020-12-09

**Authors:** Nina I. Giricheva, Sergey N. Ivanov, Anastasiya V. Ignatova, Mikhail S. Fedorov, Georgiy V. Girichev

**Affiliations:** 1 Department of Fundamental and Applied Chemistry, Ivanovo State University, 153025 Ivanovo, Russia; serg_ivan@inbox.ru (S.N.I.); nastya.ignatova2018@mail.ru (A.V.I.); fedorovms@ivanovo.ac.ru (M.S.F.); 2 Department of Physics, Ivanovo State University of Chemistry and Technology, 153000 Ivanovo, Russia; girichev@isuct.ru

**Keywords:** benzenesulfonic acids, conformer, deprotonation energy, intramolecular hydrogen bond, *ortho*-effect

## Abstract

Structural factors have been identified that determine the gas-phase acidity of *ortho*-substituted benzenesulfonic acid, 2-XC_6_H_4_–SO_3_H, (X = –SO_3_H, –COOH, –NO_2_, –SO_2_F, –C≡N, –NH_2_, –CH_3_, –OCH_3_, –N(CH_3_)_2_, –OH). The DFT/B3LYP/cc-pVTZ method was used to perform conformational analysis and study the structural features of the molecular and deprotonated forms of these compounds. It has been shown that many of the conformers may contain anintramolecular hydrogen bond (IHB) between the sulfonic group and the substituent, and the sulfonic group can be an IHB donor or an acceptor. The Gibbs energies of gas-phase deprotonation Δ_r_G^0^_298_ (kJ mol^–1^) were calculated for all compounds. It has been set that in *ortho*-substituted benzenesulfonic acids, the formation of various types of IHB is possible, having a significant effect on the Δ_r_G^0^_298_ values of gas-phase deprotonation. If the –SO_3_H group is the IHB donor, then an ion without an IHB is formed upon deprotonation, and the deprotonation energy increases. If this group is an IHB acceptor, then a significant decrease in Δ_r_G^0^_298_ of gas-phase deprotonation is observed due to an increase in IHB strength and the A^−^ anion additional stabilization. A proton donor ability comparative characteristic of the –SO_3_H group in the studied *ortho*-substituted benzenesulfonic acids is given, and the Δ_r_G^0^_298_ energies are compared with the corresponding values of *ortho*-substituted benzoic acids.

## 1. Introduction

Compounds with increased acidic properties have attracted the attention of researchers [1,2,3,4,5,6,7,8]. Among them, a special place was occupied by substituted arenesulfonic acids (ASA), which have found application in homogeneous catalysis [9,10,11,12], electrochemical technologies [13,14,15,16,17,18,19,20,21,22], and as components of ionic liquids [23,24,25].

Sulfonic groups of ASA are structural fragments responsible for proton transfer and providing electrical conductivity of proton exchange membranes. This makes it possible to use various substituted ASA as modifiers of polymer proton-exchange membranes of chemical current sources [14,15]. The branched hydrophobic chain of fluorocarbon membranes [16,17,18], membranes based on polyimides and styrenes [22], contains hydrophilic sulfonate-anionic groups, the hydration shells of which provide the membrane electrical conductivity due to the relay mechanism of proton transfer [19,20,21]. The problem of increasing the proton conductivity of polymer membranes remains actual [21,22], and the benzene sulfonic acid (BSA) molecule remains the basis for studies of the conductivity of a hydrocarbon membrane. When creating membranes with desired properties, it is necessary to understand the mechanisms and regularities of the influence of the substituted BSA structural features on the proton-donating ability of sulfonic groups and the proton-conducting ability of the corresponding hydration shells. The proton-donor properties of BSA can be controlled by introducing functional groups into it that ensure the stabilization of anionic fragments due to the intramolecular effects of delocalization of the negative charge of the anion, the formation of stable conformers with an intramolecular H-bond (IHB) [26,27,28,29], and the subsequent formation of mono- and polyhydrate complexes [15,16,17].

The results of studying the proton conductivity and computer modeling of anhydrous aromatic sulfonic acid molecules and their crystal hydrates showed that some hydrates of substituted benzenesulfonic acid have increased proton-donor properties and conductivity: phenol-2,4-di-BSA [30], 2-hydroxy-4-methyl-BSA [31], 2-sulfobenzoic [32], calixarensulfonic [33], hexasulfonic [34] acids. A distinctive feature of the molecules of the listed compounds is the presence of carboxyl, hydroxy, nitro and even second sulfonic groups in the *ortho*-position to the sulfonic group, which exhibit electronic and steric effects of a different nature.

We have previously shown that the molecules of such ASA are characterized by several non-rigid coordinates, difficulty ofconjugating *ortho*-substituents with the benzene ring, competition of orbital and steric interactions, and the formation of an IHB in a number of conformers between substituents and acidic groups –SO_3_H or –COOH. Increased proton-donating properties are inherent in ASA molecules containing an electron-withdrawing group [35,36,37,38].

It has been shown in [2,3,4,5,6,7,8] that the Gibbs free energy Δ_r_G^0^_298_ of gas-phase deprotonation (GD), can be used as a criterion for the proton-donating ability of organic acids. The value Δ_r_G^0^_298_ GD is used to build the scales of molecular acidity and to identify compounds with superacidic properties. In several works (for example [2,37,39,40]), a correlation between the energies of gas phase deprotonation and pKa values was noted. The authors [41] introduced a classification of the strength of acids, according to which an acid is considered superstrong if the Gibbs free energy of gas phase deprotonation <300 kcal/mol (1256 kJ mol^–1^). Analysis of the changes in these values for various substituted acids can identify the effects that are directly related solely to the effect of a substituent and IHB on the proton-donating ability of the compounds excluding the effect of the medium.

The presence of functional groups in BSA is the reason for the formation of conformers with different types of IHB, their different thermodynamic stability, and theirdeprotonate ability [37].

In [37], the thermodynamic characteristics of the GD of nitro-substituted BSA have been calculated. The smallest value of Δ_r_G^0^_298_ was obtained for the compound 2,4,6-(NO_2_)_3_C_6_H_2_SO_3_H, as a compound with super acidic properties [37]. Along with GD, the geometric and electronic structure of *ortho*-nitro-substituted BSAs [35,36], as well as BSA itself [42], were studied.

In the literature, the features of GD of benzoic acid (BA) as the simplest representative of substituted aromatic acids are discussed in sufficient detail [26,43,44,45,46]. The influence of the nature of substituents on the energy of GD of *meta*- and *para*-substituted BA have been considered in [43]. The ΔH_acid_ values of substituted Bas are calculated by the MP2 method increase from 1379.1/1377.5 to 1428.1/1434.8 kJ mol^−1^ (*meta*-/*para*-) and correlate with the Hammett constants σ_M_ and σ_P_ for many small substituents from –NO_2_ through the halogens and to –OH and –NH_2_. Note that, in this work, the GD energy was calculated as the difference between the energies of the most stable conformers of anions and molecular forms of acids.

For *ortho*-substituted BA, along with the factors that occur in *meta*- and *para*-substituted BA, the steric factor and the possibility of IHB formation are added [44,45]. However, IHB is absent in most 2-substituted benzoic acids, but in the compounds where H-bonds is present, for instance 2-N(CH_3_)_2_-BA and 2-OCH_3_-BA, it affects the acidity only moderately [44].

In another work [45], the effect of the *ortho*-substituent on the acidity of four *ortho*-substituted benzoic acids, 2-X-BA (X = 2-OH, 2-NH_2_, 2-COOH, and 2-SO_2_NH_2_), was considered in detail. Each of the acids has several conformers, including the conformers with IHB. It was shown that their anions are stabilized by H-bonds, and both destabilizing (2-COOH, 2-SO_2_NH_2_) and stabilizing interactions (2-OH, 2-NH_2_) are observed in acid molecules.

BSA and its substituted ones have a significantly higher deprotonation ability as compared to substituted BA. It is shown that the values Δ_r_G^0^_298_ of GD for substituted BA are ~92 kJ mol^−1^ higher than the corresponding values for substituted BSA. [37]

It is natural to expect that the formation of IHB in *ortho*-substituted BSA has its own peculiarities due to the pyramidal nature of the sulfonic group and stronger polarization of O–H bonds than those in substituted BA. Of course, it is also important that the structurally nonrigid BSA molecule exists in the form of two mirror conformers of C_1_ symmetry (enantiomers), capable of mutually transforming into each other through the transition state of C_s_ symmetry due to rotation of the –OH group around the S–O(H) bond [42]. In addition, in substituted BSA, the –SO_3_H group can occupy different spatial orientations relative to the substituent and the benzene ring [35,36,37]. In this regard, it should be expected that *ortho*-substituted BSA can form a greater number of conformers than the corresponding substituted BA, and have stronger proton-donor properties, and some of them have prospects as precursors for using high-conductivity polymer electrolytes.

**Scheme 1:**
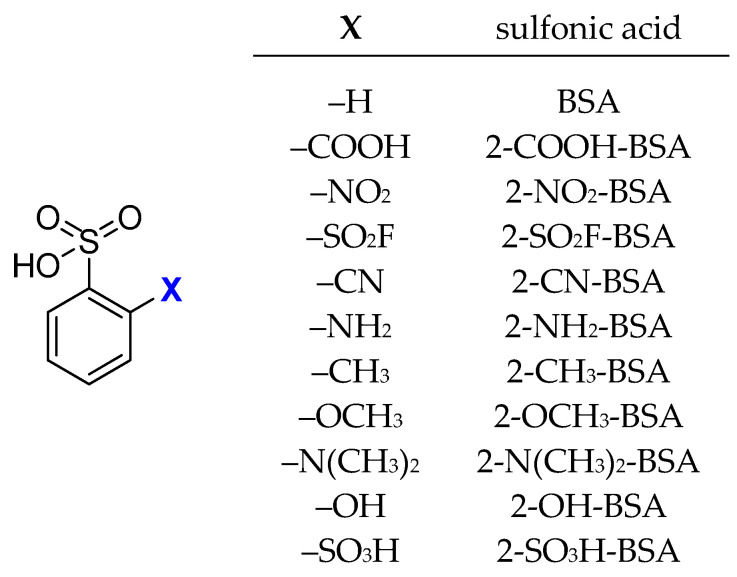
Benzene sulfonic acid and set of studied substituents.

The question of IHB’s influence on the acidic properties of *ortho*-substituted BSA is the subject of this work. Particular attention is paid to the consideration of molecules that have conformers with different types of IHB, in which the –SO_3_H group can be either a donor or an acceptor of IHB. To predict the gas-phase acidity of *ortho*-substituted BSA, the relationship between the geometric and electronic structure of molecules and deprotonation energies was studied using the example of ten *ortho*-substituents BSA of a different nature (Scheme 1).

## 2. Calculation Details

Quantum-chemical calculations were performed by the DFT/B3LYP method with the cc-pVTZ basis set [47] using Gaussian 09 package [48]. It has been shown in [37] that the use of this theoretical level gives satisfactory agreement between the calculated (1410.1 kJ mol^−1^) and experimental (1395.0(84) kJ mol^−1^) values of the Gibbs free energy Δ_r_G^0^_298_ of benzoic acid gas-phase deprotonation. The best agreement with experiment is observed in the calculations with the more costly method MP2/6-311++G** (1390.4 kJ mol^−1^). However, both methods reveal similar tendencies in the change in the Δ_r_G^0^_298_ of GD for *ortho*-, *meta*-, *para*-nitro-substituted BSA [37].

It should be noted that the structural parameters and the potential function of internal rotation for BSA, as well as the relative energy of conformers of a number of substituted BSA derivatives calculated by two methods B3LYP/cc-pVTZ and MP2/cc-pVTZ with experimental electron diffraction data, were compared [35,36,42,49,50,51]. Both methods give the same functions of the internal rotation of the sulfonic group for benzenesulfonic acid [42], moreover, for all substituted derivatives of the acid, good agreement is observed between the experimental geometric parameters and the calculated ones by both the B3LYP method and the MP2 method [35,36,42] (see Supplementary Materials, Tables S1 and S2).

Possible conformers of *ortho*-substituted BSA molecules were determined by geometric optimization (DFT/B3LYP/cc-pVTZ) of all starting structures differing in the mutual position of *ortho*-substituents, as well as using data on the conformational diversity of these compounds from [35,36,37,42].

When calculating the energy of GD, a complete optimization of the geometric parameters of both neutral AH and deprotonated A^−^ acid forms was carried out. The correspondence of the optimized geometry to the minimum on the potential energy surface was verified by calculating the vibration frequencies. For the deprotonated form, the total charge was taken as −1, and the multiplicity was 1.

For acid conformers with IHB, the Natural Bond Orbital (NBO [52]) analysis was performed for estimating a relative strength of IHB. The sum of the donor-acceptor stabilization energy E^(2)^, calculated by the 2nd-order perturbation theory, between the lone electron pairs (LP) of the atom-acceptor HB and the antibonding natural orbital σ*(O–H) is considered (Table 2 and Table S3).

The dissociation energies of gaseous acids Δ_r_E were estimated as the difference between the electronic energies E_(A_^−^_)_ of deprotonated forms of acids and the energies E_(AH)_ of their molecular forms: Δ_r_E = E_(A_^−^_)_ − E_(AH)_. In this case, we took: E_H_^+^_,0_ = 0, E_H_^+^_,298_ = E_transl_ = 3/2RT = 3.7 kJ mol^−1^, H^0^_H_^+^_,298_ = 5/2RT = 6.2 kJ mol^−1^, S^0^_H_^+^_,298_ = 108.9 J mol^−1^ K^−1^) [37,53], G^0^_H_^+^_,298_ = −26.3 kJ mol^−1^.

The values of ∆_r_H^0^_298_ and ∆_r_G^0^_298_ were calculated using Equations (1) and (2)

∆_r_H^0^_298_ = H^0^_A_^−^_,298_− H^0^_AH_,_298_ + 6.2, kJ mol^−1^, (1)

∆_r_G^0^_298_ = G^0^_A_^−^_,298_− G^0^_AH,298_ − 26.3, kJ mol^−1^ (2)

The difference between our approach to assessing the gas-phase acidity of 2X-BSA from the approaches [43,44,45] is that we take into account the conformity between the acid conformer geometric structure and its anion that forms when the proton is removed from this conformer. When discussing the strength of IHB, the geometric criteria of HB were used [54].

## 3. Conformational Diversity of *ortho*-Substituted BSA Molecules

A specific situation arises if the substituent is in the *ortho* position. All studied objects have several non-rigid torsion coordinates, which determines the presence of several conformers. In the molecules under consideration, rotation of the sulfonic acid group about the C–S bond and the –OH group about the S–O bond is possible. In the case of substituents –CH_3_, –OH, –NO_2_, –SO_2_F, –NH_2_, –COOH, their rotation about the C–X bond is possible. In the case of the –NH_2_ substituent, along with this type of rotation, pyramidal inversion is possible, and in the case of the –COOH substituent, rotation of the –OH fragment about the C–O bond is possible. Depending on the *ortho*-substituent nature of the molecules, both conformers without IHB and conformers with one or two IHBs can appear. The formation of IHB in such conformers is accompanied by a redistribution of the electron density, which has a significant effect on the characteristics of the GD process and the acidity of compounds. Therefore, hereinafter, when analyzing the conformational properties of *ortho*-substituted BSA, special attention is paid to IHB. Table 1 lists the compounds studied and shows the total number of their conformers and the number of conformers with IHB.

## 4. The Conformers Structure of Molecules of *ortho*-Substituted BSA

**Figure 1:**
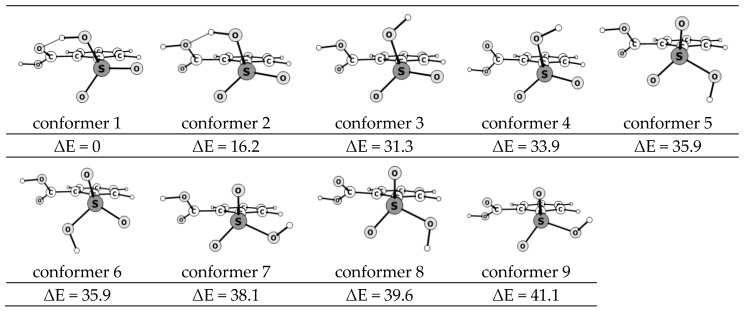
The structure of the 2-COOH-BSA conformers and their relative energies (ΔE, kJ mol^−1^).

**The 2-COOH-BSA molecule** has nine conformers (Figure 1), in which the torsion angle H–O–C=O of the –COOH group is 0°. The corresponding conformers with a torsion angle H–O–C=O of the –COOH substituent equal to 180° were not considered, since they have a higher energy (by ≈ 25 kJ mol^–1^; B3LYP/6-311++G**) [55]. In conformers 1 and 2 of 2-COOH-BSA, IHB is formed between the hydrogen atom of the –SO_3_H group and the oxygen atoms of the –COOH group (C=O or C–OH). The hydrogen bond with the carboxyl oxygen atom in conformer 1, r(O···H) = 1.735 Å, is stronger than the bond with the hydroxyl O atom in conformer 2, r(O···H) = 1.920 Å. The presence of IHB makes conformers 1 and 2 energetically more stable than other conformers in which this bond is absent.

**Figure 2:**
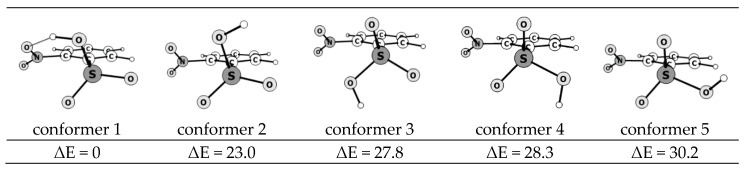
The structure of the 2-NO_2_-BSA conformers and their relative energies (kJ mol^−1^).

**The 2-NO_2_-BSAmolecule** has five conformers differing in the spatial arrangement of functional groups [35], and in one of them, an IHB is formed between the hydrogen atom of the –SO_3_H group and the oxygen atom of the –NO_2_ group, which makes this conformer energetically more favorable compared to the other conformers (Figure 2). In the conformer 1, sulfonic group is the IHB donor.

**Figure 3:**
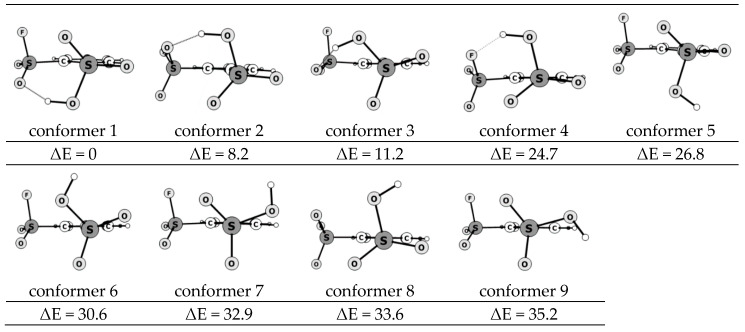
The structure of the 2-SO_2_F-BSAconformers and their relative energies (ΔE, kJ mol^−1^).

**The 2-SO_2_F-BSA molecule** has nine conformers (Figure 3), formed as a result of the –SO_2_F and –SO_3_H rotation about the corresponding C–S bonds. In conformers 1, 2, and 3, an IHB is formed between the hydrogen atom of the –SO_3_H group and the oxygen atom of the –SO_2_F group. The O···H distances are 1.825, 1.850 and 1.846 Å, respectively. The presence of IHB makes these conformers energetically more stable. In conformer 4, a less strong IHB is formed between the hydrogen atom of the –SO_3_H group and the fluorine atom of the –SO_2_F group, r(F···H) = 2.101 Å. In these four conformers, the acid group is the IHB donor. IHB is absent in other conformers.

**Figure 4:**
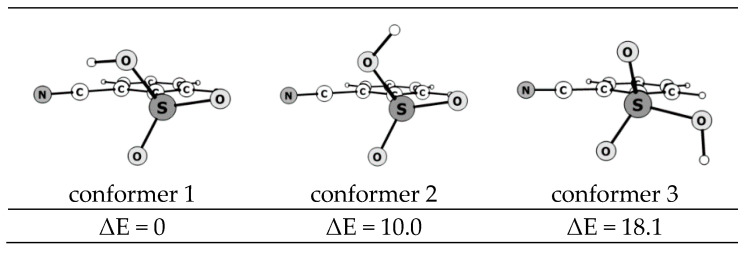
The structure of the 2-CN-BSA conformers and their relative energies (ΔE, kJ mol^−1^).

**The 2-CN-BSA molecule** has three conformers, differing in the spatial orientation of the sulfonic group (Figure 4). There is no IHB in the conformers. Note that in conformer 1, there is an interaction between the hydrogen atom of the –SO_3_H group and the nitrogen atom of the –CN group, the OH···N distance is 2.638 Å, which stabilizes the structure and makes this conformer the most stable.

**Figure 5:**
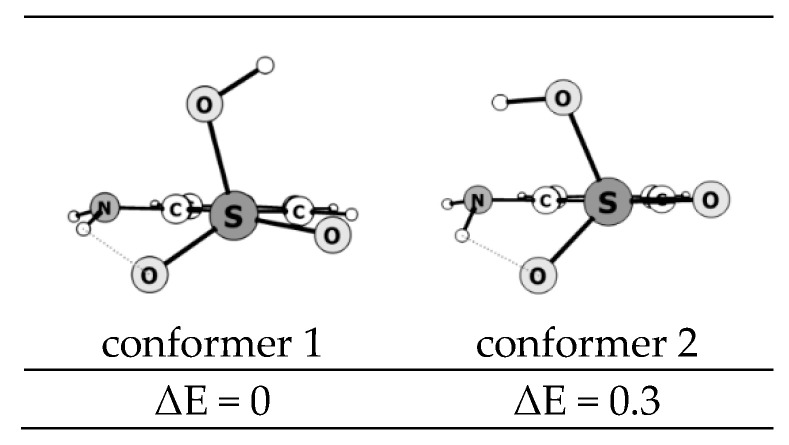
The structure of the 2-NH_2_-BSAconformers and their relative energies (ΔE, kJ mol^−1^).

**The 2-NH_2_-BSA molecule** has two conformers, the structure of which is shown in Figure 5. In both conformers of the 2-NH_2_-BSAmolecule, an IHB is observed between the hydrogen atom of the –NH_2_ group and the oxygen atom of the –SO_3_H group, where nitrogen is the donor of the hydrogen bond, and oxygen is the acceptor.

In conformer 1, an IHB is formed, which is characterized by an NH···O distance of 2.064 Å. The hydrogen atoms of the –NH_2_ group lie below the plane of the benzene ring and their position is associated with the tendency of one of the hydrogen atoms to form an IHB with the oxygen atom of the –SO_3_H group, i.e., the sulfonic group is a hydrogen bond acceptor.

In the second conformer, a less strong IHB is formed between the same atoms, which can be judged from the NH···O distance, which is 2.133 Å. However, conformer 2 is characterized by an interaction between the hydrogen atom of the –SO_3_H group and the nitrogen atom of the –NH_2_ group, the OH···N distance is 2.484 Å, which also stabilizes the structure and leads to close energies of the two conformers.

**Figure 6:**
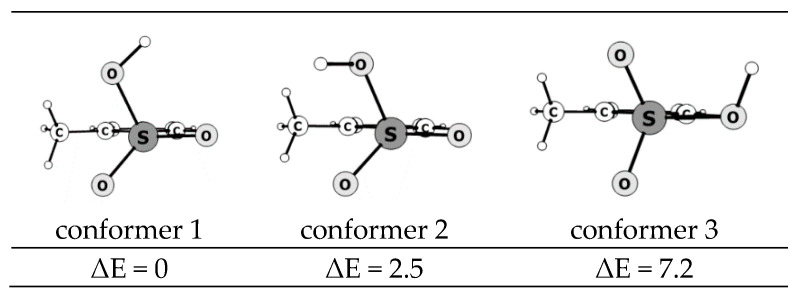
The structure of the 2-CH_3_-BSA conformers and their relative energies (ΔE, kJ mol^−1^).

**The 2-CH_3_-BSAmolecule** has three conformers (Figure 6). These conformers have close energies and differ in the sulfonic group position relative to the plane of the benzene ring. In two conformers, the S=O bond has the coplanar position relative to the benzene ring, and in the third conformer, the S–O(H) bond is coplanar to the benzene ring.

A feature of the structure of 2-CH_3_-BSA conformers is that two hydrogen atoms of the –CH_3_ group tend to be as close as possible to two oxygen atoms of the –SO_3_H group. In this case, the CH···O distance in conformers lies in the range from 2.6 to 2.7 Å. This distance is close to the sum of the van der Waals radii of oxygen and hydrogen atoms. The interaction of CH···O stabilizes the structure of each of the conformers.

**Figure 7:**
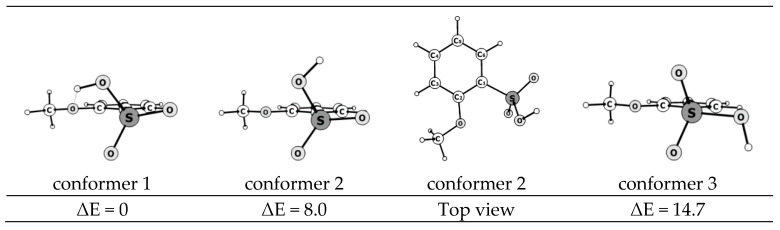
The structure of the 2-OCH_3_-BSA conformers and their relative energies (ΔE, kJ mol^−1^).

**The 2-OCH_3_-BSAmolecule** has three conformers (Figure 7).

In all conformers, the O-C bond of the –OCH_3_ group practically eclipses the C2-C3 bond of the benzene ring, which indicates the presence of an anomeric effect between the oxygen lone pair of p_π_-character and the π*-MO of the benzene ring. In conformer 1, a weak IHB is formed between the hydrogen atom of the –SO_3_H group and the oxygen atom of the –OCH_3_ group (r(O···H) = 2.176 Å), which stabilizes the structure and makes this conformer the most stable.

**Figure 8:**
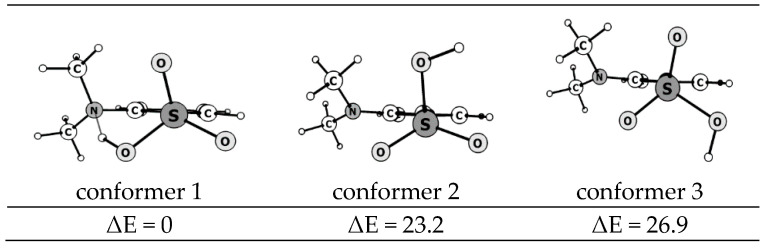
The structure of the 2-N(CH_3_)_2_-BSAconformers and their relative energies (ΔE, kJ mol^−1^).

**The 2-N(CH_3_)_2_-BSA molecule** has three conformers, the structure of which is shown in Figure 8. The nitrogen atom of the –N(CH_3_)_2_ group lies in the plane of the benzene ring, and the –CH_3_ groups are located above and below this plane. In conformer 1, an IHB is formed between the hydrogen atom of the –SO_3_H group and the nitrogen atom of the –N(CH_3_)_2_ group, r(H···N) = 1.765 Å. This conformer is the most stable.

**Figure 9:**
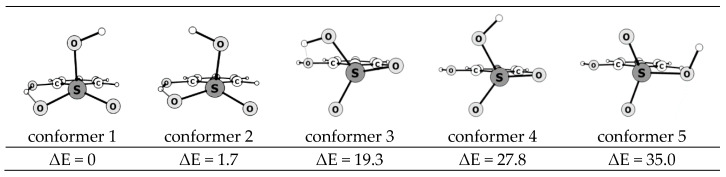
The structure of the 2-HO-BSA conformers and their relative energies (ΔE, kJ mol^−1^).

**The 2-HO-BSA molecule** has five conformers (Figure 9). Three of them have an IHB. In conformers 1 and 2, a IHB is formed between the oxygen atom of the –SO_3_H group and the hydrogen atom of the –OH group, r(O···H) is 1.820 and 1.791 Å, respectively. The sulfonic group is an acceptor of the IHB. In conformer 3, the IHB arises between the oxygen atom of the –OH group and the hydrogen atom of the –SO_3_H group, r(O···H) = 2.253 Å; the sulfonic group is a donor of the IHB. This IHB is weaker; its formation requires a greater turn of the –SO_3_H group relative to its initial position in the unsubstituted BSA. Therefore, the energy of conformer 3 is higher than the energy of conformers 1 and 2.

In conformers 4 and 5, the oxygen atoms of the –SO_3_H group are arranged so that one of the O atoms practically lies in the plane of the benzene ring. In this case, the other two oxygen atoms are almost equidistant from the oxygen atom of the –OH group. IHB is not formed in these conformers.

**Figure 10:**
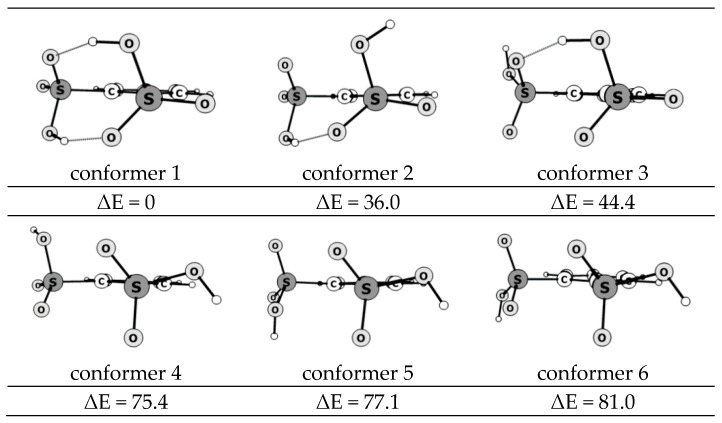
The structure of the 2-SO_3_H-BSAconformers and their relative energies (ΔE, kJ mol^−1^).

**For the 2-SO_3_H-BSA molecule**, six conformers are considered (Figure 10), which include all the main types of mutual arrangement of two sulfonic groups. Three of them contain IHBs formed between the hydrogen and oxygen atoms of neighboring sulfonic groups. Due to the formation of two strong IHBs, r(O···H) = 1.728 Å, conformer 1 is the most energetically favorable. Conformers 2 and 3, with one IHB and distances r(O···H) equal to 1.781 and 1.796 Å, respectively, have significantly higher energies (Figure 10). The relative electronic energy of conformers 4, 5, and 6, in which IHB is not formed, reaches 81.0 kJ mol^−1^. Comparison of the ΔE conformers allows us to conclude that the energy of IHB in conformers 1, 2, and 3 is not less than 37 kJ mol^−1^.

## 5. Structure of Deprotonated Forms of *ortho*-Substituted BSA

**Figure 11:**
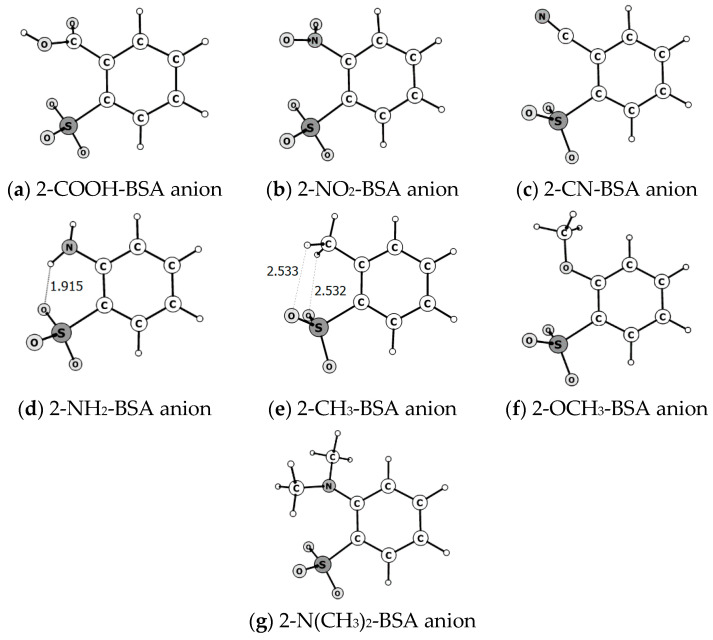
The structure of deprotonated forms of BSA (by sulfonic group), containing *ortho* substituents: –COOH, –NO_2_, –CN, –NH_2_, –CH_3_, –OCH_3_, –N(CH_3_)_2._

Despite the significant number of conformers in the ten considered molecules of *ortho*-substituted BSA, the deprotonated forms (by sulfonic group) of A^−^, seven of them are realized as single (unique) structures shown in Figure 11.

**Figure 12:**
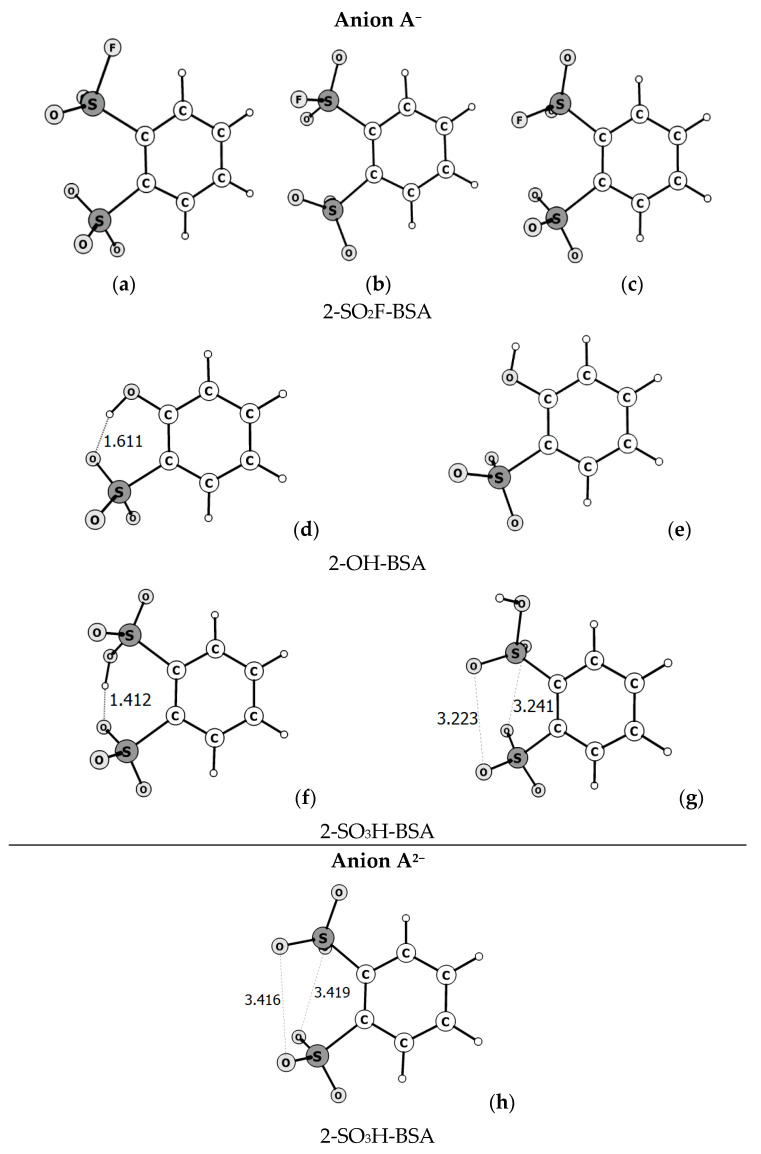
The deprotonated forms structure of A^−^acid 2-SO_2_F-BSA (**a**–**c**), 2-OH-BSA acid (**d**,**e**) and forms A^−^ (**f**,**g**) and A^2^^−^ (**h**) acid 2-SO_3_H-BSA.

At the same time, the anions of the acids 2-SO_2_F-BSA, 2-OH-BSA and 2-SO_3_H-BSA each have several conformers. The deprotonated form of 2-SO_2_F-BSA has three conformers (Figure 12a–c). The deprotonated forms A^−^ of molecules 2-OH-BSA and 2-SO_3_H-BSA have two conformers (Figure 12d,e) and (Figure 12f,g), and one of the anions of the molecules contains IHB and has a lower energy compared to anion without IHB. Figure 12h also shows the structure of the A^2−^ anion, which corresponds to the doubly deprotonated form of the 2-SO_3_H-BSA.

When a proton is removed from the –SO_3_H group, the –SO_3_^−^ fragment significantly changes its geometry, which obtains a symmetry close to C_3_. The S=O double bonds become longer, and the S–O(H) bond is shorter (by ≈ 0.2 Å) than in the initial –SO_3_H structure. All bond angles O–S–O are close to 115°, in contrast to the bond angles O=S=O ≈ 123° and O=S–O ≈104° in the –SO_3_H group.

The insignificant total natural charge +0.123 e^−^ of the –SO_3_H group in the molecular form of the acid changes to −0.65 e^−^ on the –SO_3_^−^ fragment of the deprotonated form (the example, for the conformer 1 of acid 2-OH-BSA, Figure 9; and the anion A^−^, Figure 12a).The rest of the negative charge is delocalized on the remaining part of the anion.

It is interesting to note that the IHB in deprotonated forms of A^−^ is stronger than in the corresponding acid conformers of AH (2-NH_2_-BSA, 2-NO_2_-BSA, and 2-SO_3_H-BSA, as evidenced by a longer distance r(D···H) and a shorter distances r(A···H) and r(A...D) in the anions A^−^ (Table 2). This fact was noted in [43,44] when considering the molecular and anionic forms of *ortho*-substituted BA.

**Figure 13:**
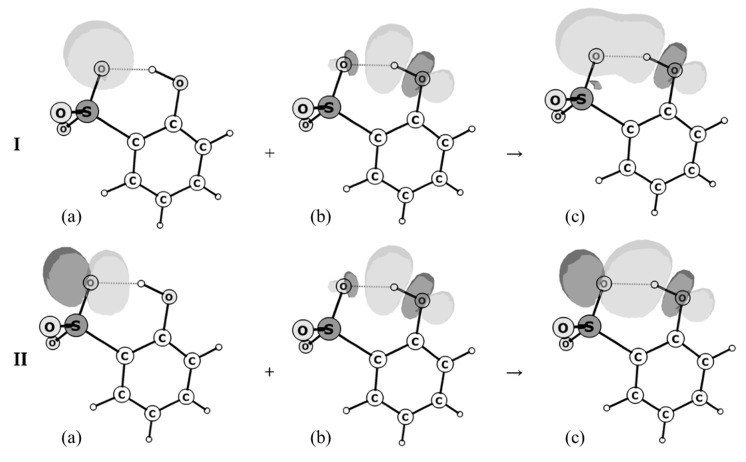
Deprotonated form of 2-OH-BSA. The natural bond orbitals and the donor-acceptor stabilization energy E^(2)^: **I** (**a**) donor LP1(O), (**b**) acceptor σ*(O‒H), (**c**) LP1(O)→σ*(O-H), E^(2)^ = 29.7 kJ mol^−1^; **II** (**a**) donor LP2(O), (**b**) acceptor σ*(O‒H), (**c**) LP2(O)→σ*(O-H), E^(2)^ = 118.5 kJ mol^−1^.

For all conformers of 2-NH_2_-BSA, 2-OH-BSA and 2-SO_3_H-BSA acids, the sum ΣE^(2)^ of the donor-acceptor stabilization energies characterized the strength of the formed IHB is presented in Table 2. As an example, the Figure 13 shows the interacting orbitals and the result of their interaction, which leads to the formation of the bonding area between O∙∙∙H atoms (deprotonated form of 2-OH-BSA).

Thus, the sum ΣE^(2)^ in the conformers of 2-NH_2_-BSA and 2-OH-BSA(1) is much lower than in the deprotonated forms of these acids (Figure 12), which, along with the geometric characteristics (Table 2) illustrates an increase in the IHB strength at the transition from the neutral to the deprotonated form (AH → A^−^).

When comparing IHB in three conformers of 2-SO_3_H-BSA acid, one may conclude that IHB in conformer 1 is stronger than in conformers 2 and 3. This is confirmed by the sum of the donor–acceptor stabilization energies (Table 2).

In the deprotonated form of the dibasic 2-SO_3_H-BSA acid, IHB is so strong that in NBO analysis the H^δ+^ stands out as an independent unit and does not form a σ(O–H) bond with any of the two O atoms, while the O1∙∙∙H∙∙∙O2 triad contains only donor–acceptor interactions between LP(O1), LP(O2) and 1s-AO(H). The fact that IHB is very strong is confirmed by the values of natural charges in the O1∙∙∙H∙∙∙O2 fragment (−0.92...+ 0.51...−1.00), which practically coincide with O1 and O2 atoms.

For hydrogen bonds of medium strength, as in the deprotonated form 2-OH-BSA, these charges in the O1–H∙∙∙O2 fragment are (−0.69...+ 0.50...−1.04).

## 6. Influence of IHB on Δ_r_G^0^_298_Gas-Phase Deprotonation Value of Different Conformers of *ortho*-Substituted BSA

In the works [44,45], the possibility of an IHB presence in the conformers of some *ortho*-substituted BA and its insignificant effect on their acidity was noted [44]. The role of the –COOH group in the IHB formation (either a donor or an acceptor of IHB) was left without discussion.

This section presents the results of calculations of the Δ_r_G^0^_298_ gas-phase deprotonationvalues for various conformers of the considered *ortho*-substituted BSA and the effect of the presence of IHB on the gas-phase acidity.

When calculating Δ_r_G^0^_298_, the correspondence of the neutral form structure of the acid AH and the anion A^−^ formed during deprotonation was determined.

For example, deprotonation of conformers 1 and 2 of the 2-OH-BSA molecule (Figure 9) leads to the formation of an anion conformer (Figure 12d), and deprotonation of conformers 3, 4, and 5 leads to another anion conformer (Figure 12e).

Table 3 shows the correspondence between the conformers of the acids AH and those anions that are formed during their deprotonation. Three types of deprotonation process can be distinguished, which differ in the presence or absence of IHB in the conformers of acids AH and the corresponding anions A^−^.

Table 4 shows the ∆_r_G^0^_298_ values for the three considered types of deprotonation processes.

## 7. The Effect of the X Substituent Nature on the Δ_r_G^0^_298_ Values

By varying the nature of the substituent, it is possible to change the value of the Gibbs free energy of the GD process.

**Scheme 2:**
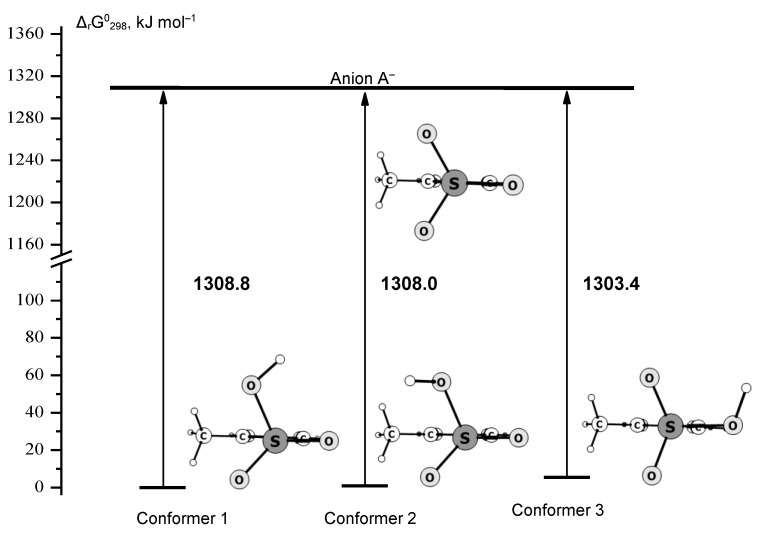
Deprotonation of 2-CH_3_-BSA conformers.

The first type of deprotonation process in the absence of a hydrogen bond will be discussed below. All molecules, except for BSA and 2-NH_2_-BSA, have several conformers without IHB (Table 1). When these conformers are deprotonated (with the exception of 2-SO_2_F-BSA), a single form of the anion appears. As a result, the Gibbs free energies GD (∆_r_G^0^_298_) of acid conformers without IHB differ by the same values as the energies of the conformers themselves (Scheme 2).

For conformers without IHB (Table 4, column 3, Type 1), there is a tendency for a decrease in ∆_r_G^0^_298_upon the introduction of electron-withdrawing *ortho*-substituents in the BSA in the sequence –COOH, –NO_2_, –SO_2_F, –C≡N, –SO_3_H.

The introduction of a weak electron-donating substituent –CH_3_ practically does not change the value of ∆_r_G^0^_298_ as compared to unsubstituted BSA, while the introduction of a stronger donor substituent –OH increases the Gibbs free energy of the GD.

Since the *ortho* effect is a combination of steric and electronic factors that cannot be separated or identified as a dominant factor, it is not possible to construct simple correlations between any characteristics of conformers without IHB and ∆_r_G^0^_298_.

A similar situation was observed in a series of *ortho*-substituted BA [43,44].

The second type of deprotonation process is shown in (Table 4, column 4, Type 2). The proton of the –SO_3_H group is involved in the formation of IHB, i.e., the –SO_3_H group is a donor to the IHB.

**Scheme 3:**
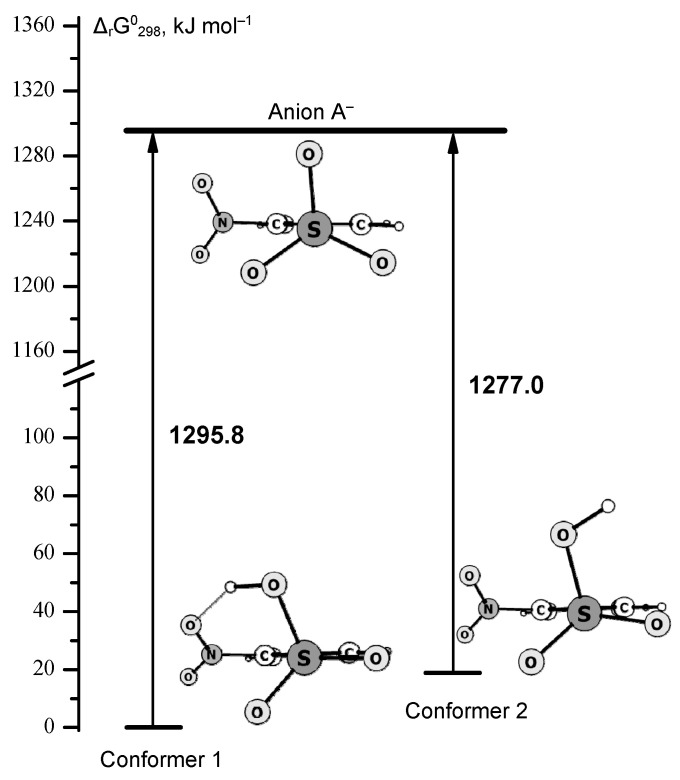
Deprotonation of 2-NO_2_-BSA conformers.

In this type of deprotonation, an increase in Δ_r_G^0^_298_ is observed (Table 4, Type 2) compared to the deprotonation of conformers without an IHB (Table 4, Type 1). The stronger the formed IHB, the more significantly the energy of the conformer decreases, and the more difficult it is to convert it into the deprotonated form (Scheme 3).

In the third type of deprotonation (Table 4, Type 3) the –SO_3_H group is an acceptor of the IHB.

Upon deprotonation of such conformers (2-NH_2_-BSA (1,2), 2-OH-BSA (1,2), 2-SO_3_H-BSA (2,3)) the IHB is retained in the anionic form A^−^, and, as noted above, its strength increases. This leads to additional stabilization of the A^−^ anion and a decrease in the energy Δ_r_G^0^_298_. At first glance, it seems that the reason for the decrease in Δ_r_G^0^_298_ GD in the conformers, where the –SO_3_H group is the IHB acceptor, is the redistribution of the electron density in this group and the change in the electronic characteristics of the S–OH fragment compared to similar characteristics in conformers without the IHB. However, it turned out that both the atomic natural charges and the S–O and O–H interatomic distances change very little. For example, in conformer 1 with the IHB and conformer 4 without the IHB, the 2-OH-BSA molecule has natural charges on the atoms q(S) = 2.348/2.346 e^−^, q(O) = −0.875/−0.873 e^−^, q(H) = 0.501/0.497 e^−^, and the distances r(S-O) = 1.625/1.625Å, r(O–H) = 0.968/0.968 Å and the valence angle S–O–H = 108.2/107.2°, respectively.

**Scheme 4:**
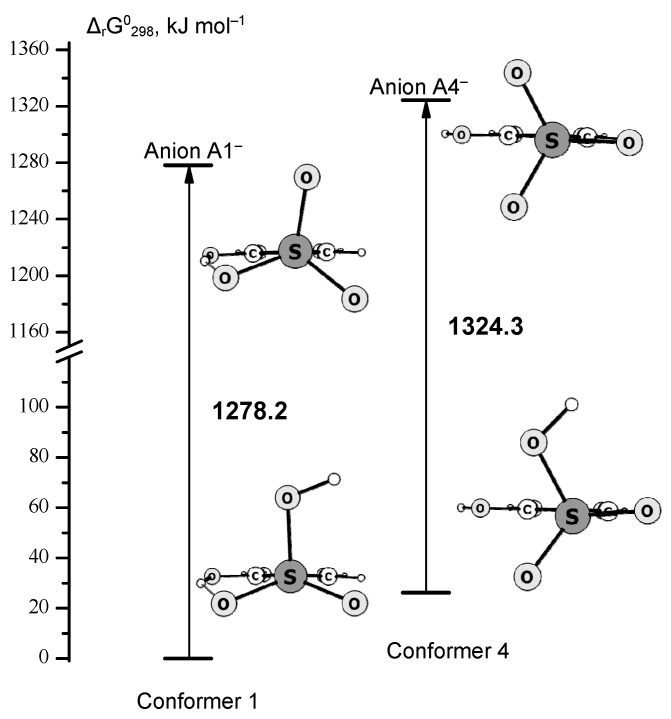
The deprotonation of 2-OH-BSA conformers (the –SO_3_H group is a acceptor of the IHB).

Therefore, the main reason for the differences in the Δ_r_G^0^_298_ GD of such conformers is the difference in the structure and energy of the anions formed on the deprotonation of the conformers. Upon deprotonation of conformer 1, the IHB is retained and, moreover, becomes stronger. The energy of this anion A1^−^ is significantly lower (Scheme 4) than the A4^−^ anion without IHB, which is formed upon deprotonation of conformer 4.

The deprotonation energy depends both on the structure of the acid conformers and on the structure of the anions that are formed on their deprotonation.

## 8. Features of 2-SO_3_H-BSA Deprotonation: The Influence of IHB on Proton Donor Properties of Diacid

The effect of the IHB on the deprotonation energy of 2-SO_3_H-BSA demonstrates the unique structural features of this acid due to the existence of a large number of various conformers (Figure 10). In conformers 1, 2 and 3, the proton of the –SO_3_H group is involved in the formation of the IHB, and in the conformers 4, 5 and 6, the IHB is not formed. For conformers 4, 5 and 6, the Δ_r_G^0^_298_ GD values for the first stage are 1279.9, 1277.8, and 1275.3 kJ mol^−1^.

**Scheme 5:**
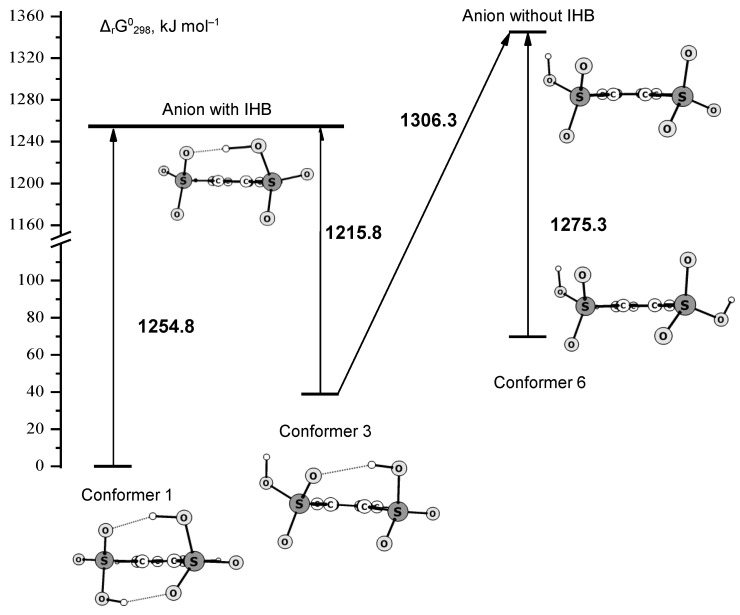
The deprotonation of 2-SO_3_H-BSA conformers in the first stage.

In conformers 2 and 3 (Scheme 5), the separation of a proton from the sulfonic group, which is an IHB acceptor (Δ_r_G^0^_298_ = 1224.2, 1215.8 kJ mol^−1^), is greatly facilitated, and the separation of a proton from another sulfonic group, which is a donor IHB, is significantly hindered (1306.3 kJ mol^−1^). Again, the reason for this trend is the IHB strengthening on the transition from conformers 2 and 3 to the A^−^anion (Figure 12f, Table 2).

In conformer 1, both H atoms of sulfonic groups participate in the formation of the IHB. The hydrogen bonds in this conformer are the strongest, which leads to a shortening of r (O···H) = 1.728 Å, compared with r (O···H) = 1.781/1.796 Å in conformers 2 and 3 with one H-bond.

Both sulfonic groups of conformer 1 are both donors and acceptors of the IHB. Therefore, for the first stage of GD, the value Δ_r_G^0^_298_ (1254.8 kJ mol^−1^) of conformer 1 is close to the average value of the deprotonation energies of the sulfonic groups—the IHB acceptor (1215.8 kJ mol^−1^) and the sulfonic group—the IHB donor (1306.3 kJ mol^−1^) in the conformer 3 (Table 4, Scheme 5).

The proton separation at the second stage of deprotonation of various conformers of 2-SO_3_H-BSA diacid leads to the formation of a dianion (Figure 12h), which has a geometric structure of symmetry C_2_. The second stage deprotonation requires more significant energy costs (Table 5).

**Scheme 6:**
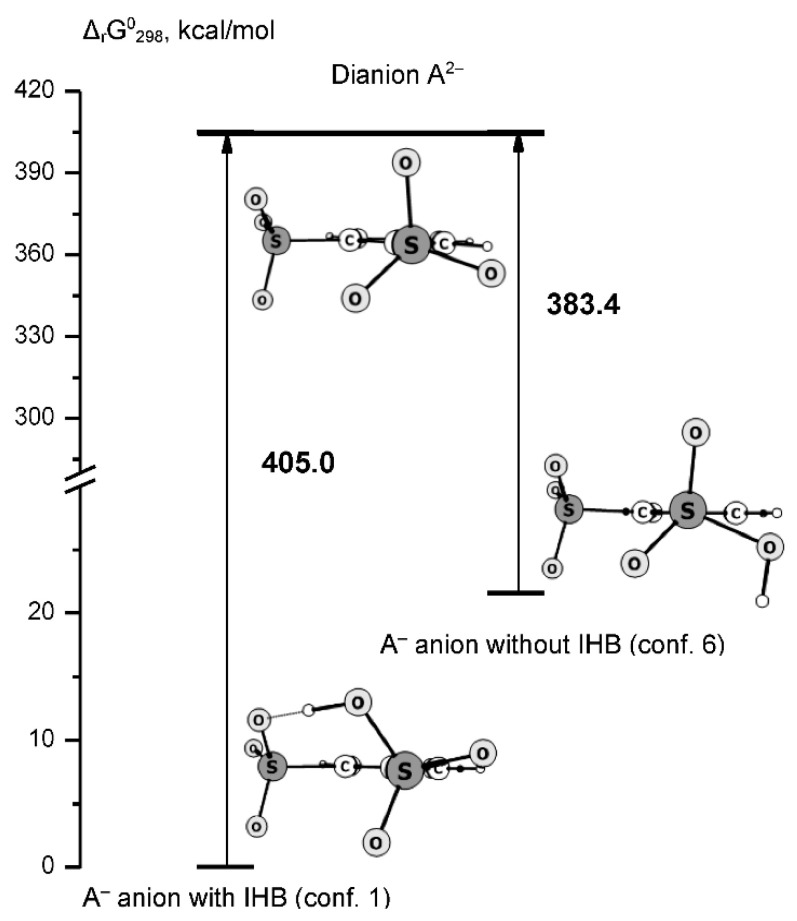
The deprotonation of 2-SO_3_H-BSA conformers in the second stage.

The deprotonation energy in two stages substantially depends on the structure of the 2-SO_3_H-BSA conformers. An interesting fact is that Δ_r_G^0^_298_ in the first stage is minimal for conformer 3, however, Δ_r_G^0^_298_ GD in the second stage is minimal for conformer 4 with a maximum value of Δ_r_G^0^_298_ GD in first stage. Upon deprotonation of the anion of A^−^ conformers 1–3 (Figure 12e, Scheme 6), the proton is removed from the sulfonic group, which is the donor of IHB, and this increases the value of Δ_r_G^0^_298_. The separation of a proton from A^−^anion without an IHB (Figure 12g, Scheme 6) requires significantly lower energy costs. As a result, the formation of the A^2^^−^ dianion, which has a single structure (Figure 12h), from the anion without the IHB (conformers 4–6) is more advantageous than that from the anion with the IHB (conformers 1–3).

Data analysis of Table 3 shows that the IHB plays a significant role in the process of deprotonation of *ortho*-substituted BSA. Acid conformers, in which IHB is present, are the most energetically favorable and predominate in the conformational mixture. In conformers of *ortho*-substituted BSA without IHB, the introduction of electron-withdrawing substituents promotes a decrease, and the introduction of electron-donor substituents promotes an increase in Δ_r_G^0^_298_ GD. However, the presence of the IHB of different type in *ortho*-substituted BSA may change this tendency common for *meta*- and *para*-substituents.

Thus, the presence of IHB in 2-OH-BSA (with an electron-donating substituent and the sulfonic group—an acceptor of IHB) increases the ability of the 2-OH-BSA conformer (1) to lose the proton, compared to the corresponding ability of the 2-SO_2_F-BSA conformer (1) (with electron withdrawing substituent and the sulfonic group—an IHB donor).

## 9. Conclusions

Using the DFT/B3LYP/cc-pVTZ method, the conformational properties of ten *ortho*-substituted BSAs (2-X-BSA, X = –SO_3_H, –COOH, –NO_2_, –SO_2_F, –C≡N, –NH_2_, –CH_3_, –OCH_3_, –N(CH_3_)_2_, –OH). The *ortho*-substituted BSAs differ from the corresponding *ortho*-substituted BAs by the presence of a larger number of conformers (up to nine for 2-COOH-BSA and 2-SO_2_F-BSA). The conformers may or may not contain the IHB between the sulfonic group and the substituent, and the sulfonic group can be a donor or an acceptor IHB.

The GD of the –SO_3_H group leads to the geometry of the –SO_3_– anion with symmetry close to C_3_. For all conformers of seven acids, the deprotonated A^−^forms have the single structure. However, the deprotonated A^−^ forms of 2-SO_2_F-BSA, 2-OH-BSA, and 2-SO_3_H-BSA have several conformers. The double-deprotonated A^2−^form of 2-SO_3_H-BSA acid has C_2_ symmetry with equivalent SO_3_^−^ groups.

The deprotonation energy of various conformers of the considered acids was calculated. It was established that the presence of the IHB in *ortho*-substituted BSAs has a significant effect on the values of Δ_r_G^0^_298_ GD. The energy of the GD depends both on the structure of the conformers of the initial acid and on the structure of the formed anion. If the –SO_3_H group is an IHB donor, then anion without IHB is formed during deprotonation, and the deprotonation energy increases. If this group is an IHB acceptor, then a significant decrease in Δ_r_G^0^_298_ GD is observed due to an increase in the strength of the IHB and additional stabilization of the A^−^ anion.

The presence of different types of IHB in 2-SO_3_H-BSA conformers affects the energy of deprotonation at the first and second stages. The value Δ_r_G^0^_298_ in the first stage of GD is minimal for conformer in which one of the sulfonic groups is a donor of IHB, and the other one is an acceptor of IHB.

The total energy of deprotonation in two stages is minimal for the 2-SO_3_H-BSA conformers without IHB.

It has been shown that the acidity of BSA derivatives in the gas phase increases when an*ortho*-substituent exhibiting donor properties of IHB with respect to the sulfone group is introduced.Therefore, the fragments containing a benzenesulfonic groups with such *ortho*-substituents into the polymer chain can be used as a precursor of high-conductivity polymer membranes.

## Figures and Tables

**Table 1:** The number of acid conformers

Compound	Total Number of Conformers ^a^	Number of Conformers with IHB ^b^
BSA	1	-
2-COOH-BSA ^c^	9	2
2-NO_2_-BSA	5	1
2-SO_2_F-BSA	9	4
2-CN-BSA	3	-
2-NH_2_-BSA	2	2
2-CH_3_-BSA	3	-
2-OCH_3_-BSA	3	1
2-N(CH_3_)_2_-BSA	3	1
2-OH-BSA	5	3
2-SO_3_H-BSA	6 ^d^	3

^a^ The conformational composition of gas phase (Boltzmann distribution, 298 K) and Cartesian coordinates of optimized geometry (DFT/B3LYP/cc-pVTZ) for acid conformers and deprotonated forms are given in ESI. ^b^ IHB—intramolecular hydrogen bond. ^c^ Only low-energy conformers with the H–O–C=O torsion angle close to 0° were considered. ^d^ The molecule has a greater number of conformers. This work considers six conformers, which include all the main types of mutual arrangement of two sulfonic groups.

**Table 2:** Geometric characteristics of IHB in conformers of AH acids and deprotonated forms A^−^ (D–IHB donor, A—IHB acceptor, interatomic distances in Å, angles in degrees, the sum of donor-acceptor stabilization energies ΣE^(2)^ in kJ mol^−1^).

Acid (Conformer)	Molecule AH					Deprotonated Form A^−^				
	r(D–H)	r(A···H)	r(A···D)	∠D-H···A	ΣE^(2)^	r(D–H)	r(A···H)	r(A···D)	∠D-H···A	ΣE^(2)^
2-NH_2_-BSA (1)	1.009	2.064	2.857	133.8	17.2	1.020	1.915	2.700	142.0	41.4
2-OH-BSA (1)	0.976	1.820	2.700	148.4	53.6	1.003	1.611	2.566	157.3	148.2
2-OH-BSA (2)	0.977	1.791	2.680	150.0	61.5					
2-SO_3_H-BSA (1)	0.992	1.728	2.670	157.0	82.1	1.061	1.412	2.466	172.0	506.6
2-SO_3_H-BSA (2)	0.983	1.781	2.697	152.1	59.5					
2-SO_3_H-BSA (3)	0.983	1.796	2.704	152.1	55.3					

**Table 3:** Conformers of acids AH and anions A^−^, which are formed under their deprotonation.

**1. The Acid Conformer AH and Conformer Anion A^−^ do not Contain IHB**		
**Acid**	**Conformer numbers**	**Anion**
2-COOH-BSA	3–9, Figure 1	Figure 11a
2-NO_2_-BSA	2–5, Figure 2	Figure 11b
2-SO_2_F-BSA	5–7, 9 and 7, Figure 3	Figure 12a,b accordingly
2-CN-BSA	1–3, Figure 4	Figure 11c
2-CH_3_-BSA	1–3, Figure 6	Figure 11e
2-OCH_3_-BSA	2,3, Figure 7	Figure 11f
2-N(CH_3_)_2_-BSA	2,3, Figure 8	Figure 11g
2-OH-BSA	4,5, Figure 9	Figure 12e
2-SO_3_H-BSA	4–6, Figure 10	Figure 12g
**2. The Acid Conformer Contains the IHB; the –SO_3_H Group is a IHB Donor**		
2-COOH-BSA	1,2, Figure 1	Figure 11a
2-NO_2_-BSA	1, Figure 2	Figure 11b
2-SO_2_F-BSA	1–4, Figure 3	Figure 12a–c accordingly
2-OCH_3_-BSA	1, Figure 7	Figure 11f
2-N(CH_3_)_2_-BSA	1, Figure 8	Figure 11g
2-OH-BSA	3, Figure 9	Figure 12e
2-SO_3_H-BSA	1, Figure 10	Figure 12f
**3. The Acid Conformer Contains the IHB. The –SO_3_H Group is an Acceptor of the IHB; Anion Conformer Preserves this Connection**		
2-NH_2_-BSA	1,2, Figure 5	Figure 11d
2-OH-BSA	1,2, Figure 9	Figure 12d
2-SO_3_H-BSA	2,3, Figure 10	Figure 12f

**Table 4:** Gibbs free energies of GD kJ mol^−1^) for conformers of *ortho*-substituted BSA with a different type of IHB (conformer numbers in parentheses).

Acid/ Type of IHB	Without IHB Type 1	With IHB	
		–SO_3_H is IHB Donor Type 2	–SO_3_H is IHB Acceptor Type 3
	∆_r_G^0^_298_	∆_r_G^0^_298_	∆_r_G^0^_298_
BSA	1313.0	-	-
2-COOH-BSA	1294.1(4) ^a^	**1320.9**(1) ^b^	-
2-NO_2_-BSA	1277.0(2)	**1295.8**(1)	-
2-SO_2_F-BSA	1268.6(5)	**1291.2**(1) 1286.2(2) 1282.8(3) 1270.7(4)	-
2-CN-BSA	1270.3(3)	-	-
2-NH_2_-BSA	-	-	**1310.0**(2) **1311.3**(1)
2-CH_3_-BSA	**1308.8**(1)	-	-
2-OCH_3_-BSA	1320.5(3)	**1332.7**(1)	-
2-N(CH_3_)_2_-BSA	1313.8(2)	**1333.1**(1)	-
2-OH-BSA	1324.3(4)	1331.4(3)	**1278.2**(1)
2-SO_3_H-BSA	1 stage 1279.9(4) 2 stage 1605.2(4)	1 stage 1306.3(3) 2 stage 1695.7(3)	1 stage 1224.2(2) 2 stage 1695.7(2)

^a^ For the conformer number, see Figure 1, Figure 2, Figure 3, Figure 4, Figure 5, Figure 6, Figure 7, Figure 8, Figure 9 and Figure 10. ^b^ The ∆_r_G^0^_298_ values for the conformers, which have the lowest energy and predominant concentration in the conformational mixture, are marked in bold.

**Table 5:** Gibbs energies of GD of different conformers 2-SO_3_H-BSA (kJ mol^−1^) in two stages.

Conformer	1	3	6
	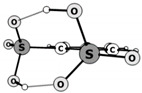	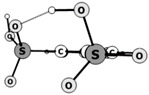	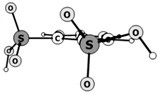
(∆_r_G^0^_298_)_I_	1254.8	1215.8 ^a^ 1306.3 ^b^	1275.3
(∆_r_G^0^_298_)_II_	1695.7	1695.7	1605.2
∑(∆_r_G^0^_298_)	2950.4	2911.5	2880.5

^a^ when a removed proton is not involved in the IHB. ^b^ when a removed proton is involved in the IHB, and the sulfonic group is IHB donor.

## Supplementary Materials

The following are available online. Table S1. Experimental (gas-phase electron diffraction—GED) and calculated (B3LYP/cc-pVTZ//MP2/cc-pVDZ//МР2/cc-pVTZ) geometries (angstroms and degrees) of unsubstituted BSA [42], Table S2. Experimental (GED) and calculated (B3LYP/cc-pVTZ//МР2/cc-pVTZ) geometries (angstroms and degrees) of 4-CH_3_-BSA molecule and conformer **I** of 3-NO_2_-BSA molecule [49], Table S3. The conformational composition of gas phase (Boltzmann distribution, 298 K) and the sum of the donor-acceptor stabilization energies (ΣE^(2)^) characterized the strength of the formed IHB in conformers with IHB, Data: Cartesian coordinates (Å) of optimized geometry (DFT/B3LYP/cc-pVTZ) of acid conformers and deprotonated forms.
