# Supplementary material for: The Effect of Intramolecular Hydrogen Bond Type on the Gas-Phase Deprotonation of ortho-Substituted Benzenesulfonic Acids. A Density Functional Theory Study
Source: Molecules. 2020 Dec 9;25(24):5806. doi: 10.3390/molecules25245806 (PMC7764180; doi:10.3390/molecules25245806)
Supplement: Supplementary file 1 [file molecules-25-05806-s001.pdf]

Supplementary Materials: The Effect of Intramolecular Hydrogen Bond Type on the Gas-Phase Deprotonation of *ortho*-Substituted Benzenesulfonic Acids. A Density Functional Theory Study

Nina I. Giricheva [1,*], Sergey N. Ivanov [1], Anastasiya V. Ignatova [1], Mikhail S. Fedorov [1] and Georgiy V. Girichev [2]

[1] Department of Fundamental and Applied Chemistry, Ivanovo State University, 153025 Ivanovo, Russia; serg_ivan@inbox.ru (S.N.I.); nastya.ignatova2018@mail.ru (A.V.I.); fedorovms@ivanovo.ac.ru (M.S.F.)

[2] Department of Physics, Ivanovo State University of Chemistry and Technology, 153000 Ivanovo, Russia; girichev@isuct.ru

* Correspondence: n.i.giricheva@mail.ru; Tel: +7-4932-373703

Table S1. Experimental (gas-phase electron diffraction – GED) and calculated (B3LYP/cc-pVTZ//MP2/cc-pVDZ//MP2/cc-pVTZ) geometries (angstroms and degrees) of unsubstituted BSA [42].

Parameter	GED, r_{h1}-structure	QC, r_e-structure
$(C–H)_{average}$	1.116(6)	1.081/1.095/1.081
$(C–C)_{average}$	1.402(4)	1.390/1.405/1.393
C–S	1.770(5)	1.786/1.781/1.767
S–O	1.623(4)	1.630/1.658/1.615
$(S=O)_{average}$	1.438(4)	1.445/1.468/1.441
O–H	0.870(17)	0.968/0.975/0.969
∠C–C(S)–C	121.6 (12)	121.7/122.1/122.0
∠S–C–C	122.9(9)	119.0/119.0/119.2
∠O–S–C	104.0 (8)	102.3/100.6/101.9
∠O=S–C	109.9(5)	109.2/109.0/108.8
∠O=S–O	106.3(10)	105.6/106.1/107.1
∠O=S=O	120.4(23)	122.1/123.4/122.7
∠H–O–S	117(6)	107.6/104.5/106.6
ϕ S–C–C–C	180.0	179.9/179.6/179.3
ϕ O–S–C–C	-97.7(44)	-95.3/-93.7/-95.8
ϕ H–O–S=O	-20 (48)	-14.1/-9.0/-19.6
ϕ H–O–S–C	96(49)	100.6/104.7/96.0

Table S2. Experimental (GED) and calculated (B3LYP/cc-pVTZ // MP2/cc-pVTZ) geometries (angstroms and degrees) of 4-CH_3-BSA molecule and conformer **I** of 3-NO_2-BSA molecule [49].

Parameter	4-CH_3-BSA GED, r_{h1}-structure	4-CH_3-BSA QC, r_e	3-NO_2-BSA GED, r_{h1}-structure	3-NO_2-BSA QC, r_e
Me $(C–H)_{average}$	1.108(5)	1.091/1.089	-	-
Ph $(C–H)_{average}$	1.099(5)	1.081/1.082	1.090(7)	1.080/1.080
Ph $(C–C)_{average}$	1.403(3)	1.391/1.394	1.395(4)	1.388/1.391
C–C(Me)	1.517(3)	1.505/1.502	-	-
C–S	1.765(5)	1.782/1.764	1.784(5)	1.791/1.774
S–O	1.618(4)	1.631/1.616	1.620(4)	1.625/1.611
$(S=O)_{average}$	1.433(4)	1.446/1.442	1.438(4)	1.443/1.440
O–H	0.968	0.968/0.968	0.968	0.968/0.969
C–N	-	-	1.477(4)	1.481/1.474
N–O	-	-	1.223(4)	1.220/1.227
∠C–C(S)–C	122.2(3)	121.1/121.6	121.6(3)	121.7/122.3
∠C–C(Me)–C	118.2(3)	118.4/118.5	-	-
∠C–C(N)–C	-	-	122.4(3)	122.3/122.8
∠ S–C–C	120.0(17)	119.6/119.1	118.9(6)	119.1/118.7
∠ O–S–C	102.8(23)	102.6/102.0	101.6(24)	101.9/101.3
∠O=S–C	109.6(10)	109.3/108.9	109.3(8)	109.0/108.7
∠O=S–O	106.9(14)	105.4/105.4	106.3(11)	107.1/107.4
∠ H–O–S	107.5	107.5/106.4	107.9	107.9/106.8
Me ∠C–C–H	106.5(26)	111.0/110.6	-	-
∠C–N–O	-	-	117.7(4)	117.4/117.2
ϕ S–C–C–C	179 (9)	179.9/179.3	175(6)	180.0/179.5
ϕ O–S–C–C	-101 (10)	-95.7/-84.6	-88(5)	-92.5/-92.4
ϕ H–O–S=O	-14.3	-14.8/-18.2	-11.0	-10.7/-14.8

Table S3. The conformational composition of gas phase (Boltzmann distribution, 298 K) and the sum of the donor-acceptor stabilization energies ($\Sigma E^{(2)}$) characterized the strength of the formed IHB in conformers with IHB.

Molecule	The conformer mole fractions (mol %)		$\Sigma E^{(2)}$ kJ mol^{-1}
**2-COOH-BSA**	Conformer 1	99.6	79.50
	Conformer 2	0.4	27.5
	Other conformers	<0.01	
**2-NO2-BSA**	Conformer 1	99.94	
	Conformer 2	0.06	42.7
	Other conformers	<0.01	
**2-SO2F-BSA**	Conformer 1	99.9	27.2
	Conformer 2	6.1	42.7
	Conformer 3	3.0	44.8
	Other conformers	<0.01	
**2-CN-BSA**	Conformer 1	95.7	
	Conformer 2	4.0	
	Conformer 3	0.3	
**2-NH_2-BSA**	Conformer 1	64.3	17.2
	Conformer 2	35.7	
**2-CH_3-BSA**	Conformer 1	55.4	
	Conformer 2	38.4	
	Conformer 3	6.2	
**2-OCH_3-BSA**	Conformer 1	91.0	
	Conformer 2	8.2	44.4
	Conformer 3	0.8	
**2-$N(CH_3)_2$-BSA**	Conformer 1	99.95	
	Conformer 2	0.04	84.6
	Conformer 3	0.01	
**2-OH-BSA**	Conformer 1	59.6	53.6
	Conformer 2	40.4	61.5
	Other conformers	<0.01	
**2-SO_3H-BSA**	Conformer 1	100	82.1
	Conformer 2	<0.01	59.5
	Conformer 3	<0.01	55.3
	Other conformers	<0.01	

Cartesian coordinates (Å) of optimized geometry (DFT/B3LYP/cc-pVTZ) of acid conformers and deprotonated forms.

2-CN-BSA

Conformer 1

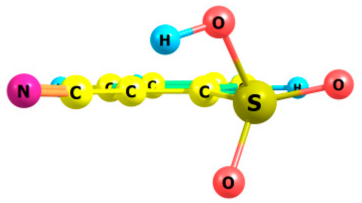

8	1.961554000	0.361248000	-1.154436000
8	1.827349000	-1.885910000	-0.207985000
8	1.910623000	0.124505000	1.313797000
16	1.470887000	-0.526149000	0.100993000
1	-0.530860000	-2.512595000	-0.061864000
6	-0.323000000	-0.393851000	0.002956000
6	-1.054907000	-1.569037000	-0.033653000
6	-0.974180000	0.850136000	0.026596000
6	-2.370182000	0.886770000	0.019332000
6	-3.099577000	-0.293777000	-0.006483000
6	-2.445347000	-1.517622000	-0.035926000
1	2.101066000	1.275361000	-0.856622000
1	-2.870951000	1.844031000	0.030653000
1	-3.012547000	-2.437497000	-0.062568000
1	-4.179735000	-0.253428000	-0.008940000
6	-0.245641000	2.078724000	0.017052000
7	0.348519000	3.065100000	-0.029542000

Conformer 2

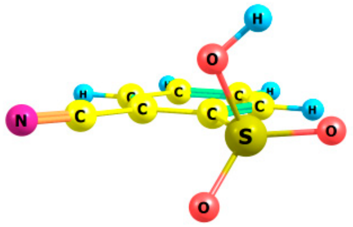

8	-1.941754000	0.335322000	1.235211000
8	-1.942374000	-1.797677000	-0.020463000
8	-1.925136000	0.428548000	-1.211785000
16	-1.508405000	-0.420448000	-0.130126000
1	0.359189000	-2.523672000	-0.031857000
6	0.286372000	-0.394043000	-0.028676000
6	0.942562000	-1.615365000	-0.015232000
6	1.011002000	0.809056000	-0.012812000
6	2.407278000	0.748750000	0.019969000
6	3.061350000	-0.475085000	0.029931000
6	2.332324000	-1.655799000	0.012322000
1	-2.142965000	-0.339190000	1.900321000
1	2.967729000	1.672192000	0.036879000
1	2.838950000	-2.610697000	0.018631000
1	4.141831000	-0.501975000	0.051549000
6	0.396564000	2.099931000	-0.008594000
7	-0.025838000	3.170900000	0.014477000

Conformer 3

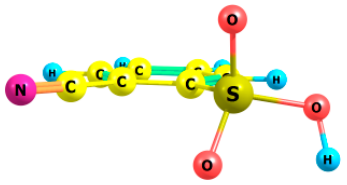

8	-2.019022000	-1.720025000	-0.191647000
8	-1.956528000	0.574383000	-1.104469000
8	-1.908192000	0.163320000	1.385842000
16	-1.526761000	-0.189795000	0.045270000
1	0.216491000	-2.509898000	0.046791000
6	0.263655000	-0.374028000	-0.001794000
6	0.844416000	-1.633484000	0.021190000
6	1.065830000	0.779112000	-0.019884000
6	2.456200000	0.632549000	-0.028307000
6	3.032927000	-0.628416000	-0.013439000
6	2.229115000	-1.759341000	0.013735000
1	-2.338508000	-1.779604000	-1.104082000
1	3.071986000	1.520035000	-0.044803000
1	2.673733000	-2.744571000	0.030715000
1	4.109578000	-0.724209000	-0.020604000
6	0.532694000	2.105879000	-0.019270000
7	0.173686000	3.199989000	-0.009075000

Anion

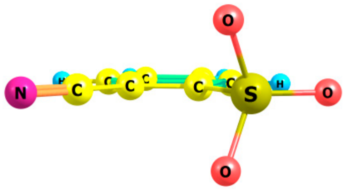

8	1.929229000	0.403386000	-1.239886000
8	2.022345000	-1.737876000	0.000061000
8	1.928612000	0.403051000	1.240348000
16	1.619480000	-0.321400000	0.000029000
1	-0.196699000	-2.509964000	-0.000593000
6	-0.217265000	-0.395415000	-0.000196000
6	-0.837612000	-1.639811000	-0.000378000
6	-1.018634000	0.756567000	0.000015000
6	-2.415768000	0.636218000	0.000267000
6	-3.016778000	-0.612401000	0.000146000
6	-2.222090000	-1.753825000	-0.000231000
1	-3.017159000	1.535296000	0.000546000
1	-2.681800000	-2.734738000	-0.000365000
1	-4.096246000	-0.691817000	0.000332000
6	-0.497913000	2.092309000	0.000000000
7	-0.229271000	3.213326000	-0.000330000

2-NO2-BSA

Conformer 1

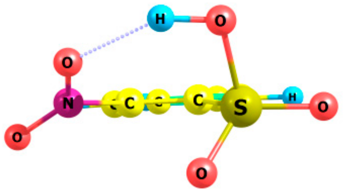

6	0.109232000	-0.626981000	0.019444000
6	0.512155000	-1.955319000	0.047972000
6	1.090367000	0.369402000	0.015845000
6	1.862580000	-2.284740000	0.088519000
6	2.438632000	0.046436000	0.031867000
6	2.824929000	-1.285881000	0.078040000
1	-0.247284000	-2.722371000	0.023282000
1	2.156140000	-3.324687000	0.117742000
7	0.777645000	1.815053000	0.032135000
8	-0.181017000	2.199597000	0.698235000
8	1.525133000	2.552275000	-0.574483000
16	-1.668750000	-0.333031000	-0.172093000
1	3.167166000	0.842420000	0.014775000
1	3.876004000	-1.536098000	0.103596000
8	-2.076472000	0.306549000	1.235565000
1	-1.612739000	1.166888000	1.295055000
8	-2.320876000	-1.615995000	-0.219113000
8	-1.835539000	0.635007000	-1.229706000

Conformer 2

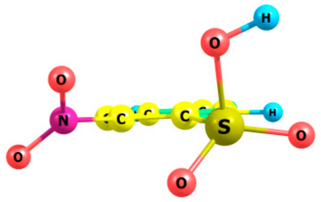

6	0.106100000	-0.608321000	0.017744000
6	0.467547000	-1.950471000	0.032238000
6	1.112386000	0.359474000	0.026942000
6	1.806801000	-2.319345000	0.082093000
6	2.448496000	-0.003703000	0.045696000
6	2.796391000	-1.347472000	0.086157000
1	-0.310236000	-2.697037000	-0.020520000
1	2.070114000	-3.367531000	0.100747000
7	0.830457000	1.812500000	0.063442000
8	-0.048169000	2.187971000	0.815603000
8	1.538325000	2.525030000	-0.627488000
16	-1.652511000	-0.258644000	-0.201813000
1	3.198553000	0.772472000	0.031072000
1	3.839775000	-1.628496000	0.113747000
8	-2.126583000	0.223721000	1.265474000
1	-2.663739000	-0.491682000	1.637220000
8	-2.314122000	-1.524874000	-0.449389000
8	-1.791177000	0.848415000	-1.107023000

Conformer 3

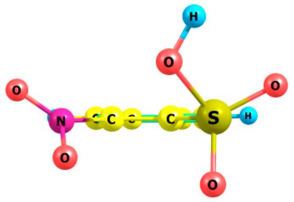

6	-0.117357000	-0.611340000	0.052315000
6	-0.514484000	-1.944769000	0.046450000
6	-1.100604000	0.379847000	0.038889000
6	-1.862234000	-2.281056000	0.057444000
6	-2.446163000	0.047638000	0.011119000
6	-2.828650000	-1.285713000	0.038252000
1	0.243258000	-2.713060000	0.015267000
1	-2.150375000	-3.322834000	0.065919000
7	-0.788893000	1.826982000	0.064968000
8	-1.488423000	2.548678000	-0.628526000
8	0.109062000	2.193479000	0.796968000
16	1.675963000	-0.356915000	0.040645000
1	-3.175225000	0.842491000	-0.028701000
1	-3.878891000	-1.541124000	0.035538000
8	1.843850000	0.865105000	-1.007467000
1	2.131318000	0.481490000	-1.849340000
8	2.252253000	-1.529031000	-0.588647000
8	2.127473000	0.090165000	1.326349000

Anion

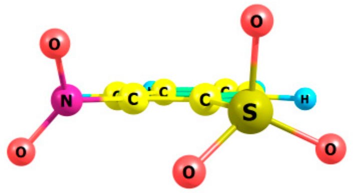

6	0.063971000	-0.606925000	0.033801000
6	0.457806000	-1.944540000	0.046712000
6	1.074531000	0.347676000	0.026405000
6	1.796848000	-2.308136000	0.070462000
6	2.421932000	0.005917000	0.014078000
6	2.786976000	-1.330719000	0.052698000
1	-0.325737000	-2.688091000	0.009185000
1	2.069470000	-3.356160000	0.086405000
7	0.802686000	1.805350000	0.047700000
8	0.206134000	2.255737000	1.004448000
8	1.284070000	2.467524000	-0.863739000
16	-1.747607000	-0.246295000	-0.062012000
1	3.165301000	0.789555000	-0.025454000
1	3.834096000	-1.604261000	0.056465000
8	-2.144446000	-0.049556000	1.336288000
8	-2.283234000	-1.471884000	-0.677350000
8	-1.814101000	0.946003000	-0.916302000

2-NH2-BSA

Conformer 1

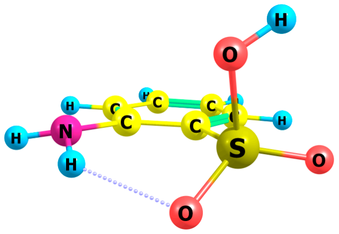

1	-0.518059000	-2.307155000	0.337049000
1	1.018638000	-3.016559000	0.140596000
7	0.419778000	-2.226889000	-0.025632000
1	4.106642000	0.412227000	-0.107126000
1	0.294305000	2.345304000	0.144523000
8	-1.952375000	-0.937061000	0.907493000
8	-1.990713000	1.532660000	0.395804000
8	-1.931128000	-0.180249000	-1.396630000
16	-1.501358000	0.197274000	0.128196000
6	0.904145000	1.456346000	0.077252000
6	0.271139000	0.213176000	0.068710000
6	1.008694000	-0.991139000	0.011516000
6	2.408572000	-0.869452000	-0.057611000
6	3.026729000	0.365054000	-0.057769000
6	2.282442000	1.542545000	0.014673000
1	-2.007941000	0.643026000	-1.899635000
1	3.002881000	-1.773141000	-0.107322000
1	2.770203000	2.506154000	0.026235000

Conformer 2

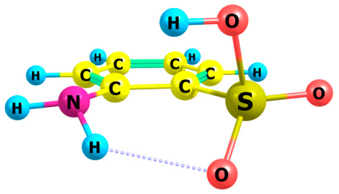

8	-1.849561000	0.328986000	1.369728000
8	-1.962564000	0.698493000	-1.083317000
16	-1.498651000	-0.265035000	-0.097541000
6	0.279607000	-0.257562000	-0.086221000
6	0.957085000	-1.470230000	-0.070765000
6	2.341977000	-1.486550000	-0.003931000
6	0.961169000	0.972257000	-0.046314000
6	2.356029000	0.930235000	0.036459000
6	3.031983000	-0.279882000	0.058609000
8	-1.995976000	-1.615178000	-0.091950000
7	0.262975000	2.179128000	-0.012121000
1	-1.564860000	1.258212000	1.394863000
1	0.385073000	-2.386055000	-0.101952000
1	2.874139000	-2.426622000	0.006738000
1	2.908297000	1.860826000	0.075146000
1	4.112202000	-0.278825000	0.117656000
1	0.849561000	2.995376000	-0.087264000
1	-0.529100000	2.215720000	-0.642389000

Anion

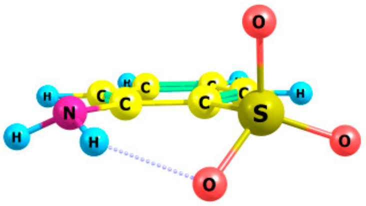

8	-1.940707000	0.021448000	1.420365000
8	-1.936631000	0.958604000	-0.864554000
16	-1.582470000	-0.191533000	0.009837000
6	0.244074000	-0.215370000	-0.009112000
6	0.895172000	-1.439755000	-0.025924000
6	2.284707000	-1.526778000	-0.011510000
6	0.996045000	0.979217000	0.010549000
6	2.396399000	0.879082000	0.019689000
6	3.031381000	-0.354062000	0.012399000
8	-1.986199000	-1.495436000	-0.537704000
7	0.377816000	2.217845000	0.053404000
1	0.280475000	-2.328633000	-0.067265000
1	2.773245000	-2.492841000	-0.025056000
1	2.983962000	1.791274000	0.035753000
1	4.114611000	-0.395275000	0.019130000
1	0.926867000	2.970757000	-0.328151000
1	-0.582724000	2.183398000	-0.287038000

2-SO3H-BSA

Conformer 1

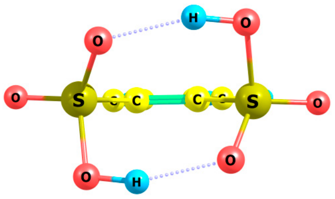

1	2.471321000	1.926791000	-0.051533000
8	3.097078000	-0.379267000	-0.178123000
8	1.136120000	-1.628211000	-1.099344000
8	1.511076000	-1.410879000	1.346966000
16	1.713566000	-0.753586000	-0.080128000
6	-0.701276000	0.742512000	0.012996000
6	0.702246000	0.741531000	-0.013262000
6	1.392005000	1.945132000	-0.026016000
6	0.694791000	3.149025000	-0.012453000
6	-0.690522000	3.149964000	0.012496000
6	-1.389383000	1.947000000	0.025819000
1	0.557680000	-1.673612000	1.428125000
1	1.242255000	4.081047000	-0.022548000
1	-1.236721000	4.082727000	0.022690000
1	-0.559370000	-1.673112000	-1.427592000
8	-1.512663000	-1.409860000	-1.346545000
16	-1.714641000	-0.751664000	0.080080000
8	-3.097723000	-0.375504000	0.177568000
8	-1.138439000	-1.626402000	1.099847000
1	-2.468729000	1.930145000	0.051192000

Conformer 2

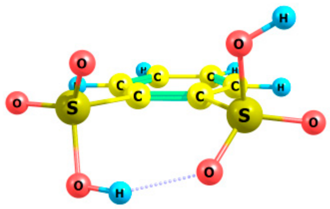

1	-2.513677000	1.866107000	0.065515000
8	-3.137848000	-0.376749000	0.135049000
8	-1.212236000	-1.639334000	1.143702000
8	-1.536597000	-1.465803000	-1.321332000
16	-1.759489000	-0.794979000	0.108451000
6	0.667752000	0.732358000	-0.020447000
6	-0.735682000	0.703388000	0.018212000
6	-1.435080000	1.902390000	0.036056000
6	-0.760933000	3.118667000	0.019687000
6	0.623071000	3.143760000	-0.020013000
6	1.336458000	1.951227000	-0.044876000
1	-0.611367000	-1.799465000	-1.351066000
1	-1.325265000	4.040699000	0.034409000
1	1.156204000	4.083871000	-0.039276000
1	2.564935000	-1.049053000	1.802784000
8	1.734148000	-1.310020000	1.377461000
16	1.739972000	-0.715959000	-0.116712000
8	3.085089000	-0.267346000	-0.400435000
8	1.124145000	-1.720070000	-0.960987000
1	2.414327000	1.956678000	-0.099565000

Conformer 3

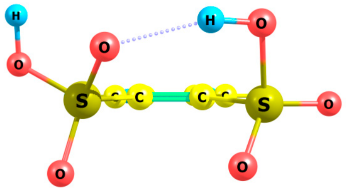

1	-2.512495000	1.888498000	-0.064189000
8	-3.159692000	-0.358774000	0.087805000
8	-1.254147000	-1.591381000	1.178466000
8	-1.533149000	-1.508966000	-1.294604000
16	-1.783323000	-0.782239000	0.105952000
6	0.653929000	0.709843000	0.016003000
6	-0.748418000	0.703685000	-0.007644000
6	-1.433216000	1.911476000	-0.046455000
6	-0.742401000	3.117023000	-0.046490000
6	0.642562000	3.120721000	-0.004437000
6	1.340372000	1.919716000	0.025219000
1	-0.622403000	-1.878247000	-1.270916000
1	-1.291974000	4.047524000	-0.073946000
1	1.189444000	4.053107000	0.004401000
1	2.418879000	1.922540000	0.048610000
8	1.111825000	-1.797700000	-0.811562000
16	1.659005000	-0.791849000	0.092627000
8	1.988504000	-1.095356000	1.453443000
8	3.036563000	-0.297034000	-0.601320000
1	3.011433000	-0.549113000	-1.536227000

Anion with IHB

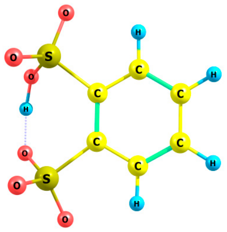

1	2.437243000	1.895352000	-0.066903000
8	3.089982000	-0.288681000	-0.454477000
8	1.188864000	-1.752311000	-0.937774000
8	1.776649000	-1.245472000	1.416639000
16	1.801871000	-0.782692000	0.034169000
6	-0.711263000	0.696755000	0.008686000
6	0.692659000	0.691119000	-0.008287000
6	1.357986000	1.914829000	-0.029228000
6	0.664492000	3.117454000	-0.016931000
6	-0.722538000	3.114604000	0.000952000
6	-1.403454000	1.905201000	0.005462000
1	1.211361000	4.051934000	-0.029723000
1	-1.277368000	4.044065000	0.002079000
1	-0.218765000	-1.777804000	-1.034608000
8	-1.268160000	-1.649148000	-1.123980000
16	-1.780263000	-0.777497000	0.066718000
8	-3.127623000	-0.344448000	-0.268991000
8	-1.570044000	-1.440711000	1.337761000
1	-2.482823000	1.875877000	-0.002390000

Anion without IHB

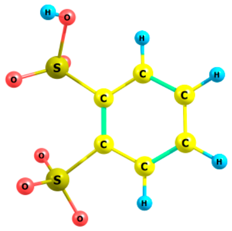

1	2.511569000	1.836994000	0.043831000
8	1.140030000	-1.762536000	-0.921822000
8	1.864844000	-1.272153000	1.408559000
8	3.116748000	-0.339602000	-0.502769000
16	1.834031000	-0.857890000	0.001407000
6	-0.638427000	0.679368000	-0.004800000
6	0.759770000	0.651460000	0.018400000
6	1.431574000	1.874458000	0.042981000
6	0.751887000	3.082141000	0.030847000
6	-0.636656000	3.094000000	-0.016523000
6	-1.329192000	1.893500000	-0.038318000
1	1.305725000	4.012850000	0.047729000
1	-1.183119000	4.028005000	-0.041737000
1	-2.406713000	1.893678000	-0.074340000
8	-1.266988000	-1.817670000	0.878264000
16	-1.669453000	-0.815308000	-0.078834000
8	-1.992049000	-1.118369000	-1.450583000
8	-3.091174000	-0.216696000	0.537028000
1	-3.065716000	-0.433700000	1.478405000

2-OH-BSA

Conformer 1

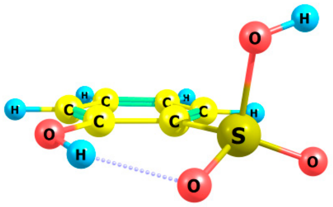

8	-1.928181000	-0.129605000	1.442024000
8	-2.021934000	-1.380870000	-0.689580000
8	-1.906766000	1.142621000	-0.629462000
16	-1.498649000	-0.157661000	-0.124745000
1	0.290501000	-2.337998000	-0.180089000
6	0.269652000	-0.201073000	-0.079030000
6	0.897896000	-1.448401000	-0.094033000
6	1.018138000	0.989232000	-0.010937000
6	2.410540000	0.880786000	0.072146000
6	3.023690000	-0.357530000	0.071872000
6	2.274645000	-1.531969000	-0.019252000
1	-2.144743000	-1.033367000	1.713039000
1	2.984574000	1.794929000	0.125621000
1	2.762133000	-2.495825000	-0.036442000
1	4.102487000	-0.411746000	0.131925000
8	0.491555000	2.223895000	-0.020244000
1	-0.461319000	2.171979000	-0.224630000

Conformer 2

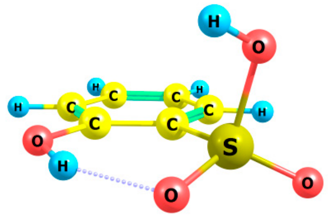

8	-1.939046000	-0.374474000	1.428307000
8	-1.996584000	-1.342778000	-0.803282000
8	-1.932700000	1.165114000	-0.505740000
16	-1.499433000	-0.178749000	-0.122925000
1	0.296145000	-2.338498000	-0.120978000
6	0.270490000	-0.199754000	-0.075176000
6	0.902165000	-1.445924000	-0.062639000
6	1.016589000	0.992648000	-0.030751000
6	2.409423000	0.890169000	0.049279000
6	3.025668000	-0.346265000	0.075434000
6	2.278670000	-1.523989000	0.013566000
1	-2.055693000	0.498621000	1.829739000
1	2.981149000	1.806834000	0.079693000
1	2.768005000	-2.487010000	0.021316000
1	4.104719000	-0.396705000	0.134084000
8	0.486416000	2.228451000	-0.060439000
1	-0.466101000	2.164925000	-0.266104000

Conformer 3

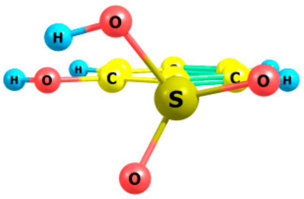

8	1.939079000	0.682080000	-1.139897000
8	1.982566000	-1.587338000	-0.253984000
8	1.930139000	0.371117000	1.325459000
16	1.516928000	-0.270079000	0.097669000
1	-0.376175000	-2.379189000	-0.038998000
6	-0.269267000	-0.253213000	0.005816000
6	-0.949367000	-1.463835000	-0.019927000
6	-0.973978000	0.954081000	0.012389000
6	-2.364020000	0.930310000	0.006985000
6	-3.038798000	-0.283594000	-0.005040000
6	-2.337605000	-1.482885000	-0.022214000
1	1.743792000	1.597338000	-0.882382000
1	-2.914700000	1.863427000	0.007970000
1	-2.864809000	-2.425741000	-0.038941000
1	-4.120258000	-0.287055000	-0.006888000
8	-0.261970000	2.118624000	0.002185000
1	-0.859005000	2.871443000	0.058378000

Anion with IHB

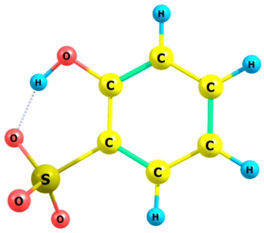

8	-1.931807000	-0.428340000	1.417720000
8	-2.010127000	-1.238529000	-0.918637000
8	-1.907308000	1.191774000	-0.451249000
16	-1.573968000	-0.193330000	0.013717000
1	0.306017000	-2.332301000	-0.082032000
6	0.244511000	-0.210332000	-0.029987000
6	0.904942000	-1.432142000	-0.041290000
6	0.988592000	0.983881000	-0.007576000
6	2.386983000	0.908535000	0.023641000
6	3.029320000	-0.319804000	0.026354000
6	2.292560000	-1.501251000	-0.012045000
1	2.945773000	1.835556000	0.039728000
1	2.792037000	-2.461643000	-0.020363000
1	4.112326000	-0.354762000	0.050055000
8	0.413311000	2.199457000	-0.022598000
1	-0.566646000	2.038207000	-0.163340000

Anion without IHB

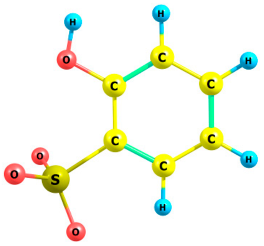

8	-1.958479000	0.560876000	1.240343000
8	-1.993533000	-1.577094000	-0.000791000
8	-1.958497000	0.562184000	-1.239568000
16	-1.615078000	-0.150933000	0.000008000
1	0.226896000	-2.311216000	-0.000066000
6	0.223540000	-0.202947000	0.000008000
6	0.861890000	-1.436096000	-0.000018000
6	1.014488000	0.954201000	0.000061000
6	2.406208000	0.848729000	-0.000028000
6	3.025379000	-0.396144000	-0.000005000
6	2.250806000	-1.547388000	0.000032000
1	3.005302000	1.754927000	-0.000108000
1	2.718552000	-2.524001000	0.000005000
1	4.107080000	-0.456865000	-0.000016000
8	0.409944000	2.176278000	0.000024000
1	1.094070000	2.852013000	-0.000318000

2-CH3-BSA

Conformer 1

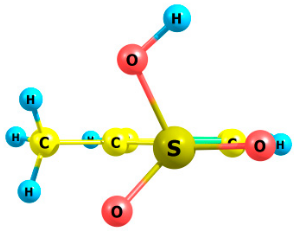

8	-1.937088000	0.428462000	1.289906000
8	-1.952720000	0.679407000	-1.141868000
8	-1.957702000	-1.617188000	-0.104752000
16	-1.499133000	-0.242763000	-0.131679000
1	0.319173000	-2.361179000	-0.076033000
1	4.124640000	-0.398766000	0.072580000
6	0.366499000	2.340517000	-0.014577000
6	0.290611000	-0.224590000	-0.042660000
6	0.920258000	-1.465037000	-0.041321000
6	1.010910000	0.980797000	-0.006626000
6	2.400812000	0.871723000	0.039003000
6	3.044005000	-0.360070000	0.039741000
6	2.305791000	-1.534103000	-0.001958000
1	-2.089340000	-0.292084000	1.917989000
1	1.121385000	3.114098000	0.111895000
1	-0.366585000	2.440422000	0.785684000
1	-0.163515000	2.517579000	-0.949796000
1	2.988770000	1.779613000	0.072816000
1	2.798365000	-2.496342000	-0.004182000

Conformer 2

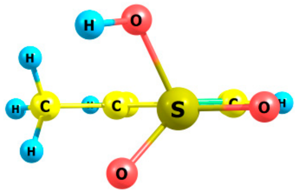

8	-1.887372000	0.383469000	1.325790000
8	-2.002789000	0.613347000	-1.130335000
8	-1.914345000	-1.664822000	-0.087679000
16	-1.492947000	-0.285115000	-0.110683000
1	0.348780000	-2.362811000	0.001324000
1	4.131968000	-0.352896000	0.043735000
6	0.344425000	2.340045000	-0.039980000
6	0.294417000	-0.227797000	-0.025349000
6	0.939905000	-1.459800000	0.001446000
6	1.000826000	0.986177000	-0.025745000
6	2.392350000	0.895229000	0.001164000
6	3.050589000	-0.328132000	0.025393000
6	2.326166000	-1.511261000	0.026294000
1	-2.572577000	1.044406000	1.148130000
1	1.098306000	3.122861000	-0.092710000
1	-0.242006000	2.498079000	0.866635000
1	-0.332897000	2.446258000	-0.885484000
1	2.969535000	1.810609000	0.001933000
1	2.830022000	-2.467375000	0.045826000

Conformer 3

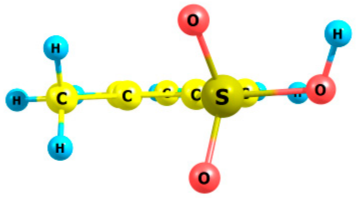

8	1.996184000	-1.597626000	-0.027234000
8	1.915519000	0.466115000	1.311693000
8	1.957426000	0.573689000	-1.200090000
16	1.502307000	-0.048348000	0.029626000
1	-0.247052000	-2.347345000	0.056312000
1	-4.114978000	-0.520309000	-0.021285000
6	-0.450037000	2.349970000	-0.021961000
6	-0.285344000	-0.205887000	0.005256000
6	-0.869412000	-1.467191000	0.025491000
6	-1.052925000	0.970528000	-0.014933000
6	-2.438058000	0.813099000	-0.026435000
6	-3.036230000	-0.440897000	-0.012778000
6	-2.253027000	-1.585002000	0.015526000
1	2.325027000	-1.755300000	-0.924114000
1	-1.234043000	3.104278000	-0.007209000
1	0.192095000	2.504305000	0.845718000
1	0.165764000	2.508072000	-0.907529000
1	-3.057968000	1.699939000	-0.044323000
1	-2.708590000	-2.565233000	0.032460000

Anoin

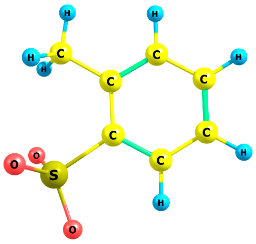

8	-1.933985000	0.514873000	1.237521000
8	-1.962959000	-1.629211000	-0.000121000
8	-1.934687000	0.515185000	-1.237129000
16	-1.579279000	-0.206841000	0.000046000
1	0.289072000	-2.342139000	-0.000140000
1	4.123530000	-0.398889000	0.000062000
6	0.336848000	2.320482000	-0.000020000
6	0.253843000	-0.223489000	-0.000216000
6	0.902663000	-1.452219000	-0.000117000
6	0.998565000	0.968684000	-0.000177000
6	2.390500000	0.871366000	-0.000069000
6	3.040561000	-0.359085000	0.000046000
6	2.292245000	-1.527897000	0.000021000
1	1.083009000	3.117115000	-0.001704000
1	-0.314114000	2.424072000	-0.868509000
1	-0.311160000	2.425308000	0.870563000
1	2.976627000	1.784089000	-0.000077000
1	2.783192000	-2.493920000	0.000088000

2-N(CH3)2-BSA

Conformer 1

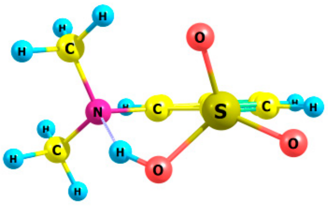

8	1.619570000	-1.080785000	-1.081271000
8	2.039886000	-0.470575000	1.294976000
8	2.653496000	1.084414000	-0.566658000
16	1.762755000	0.094331000	-0.012347000
1	0.817771000	2.746042000	0.049416000
6	0.083492000	0.744264000	0.008070000
6	-0.065165000	2.123431000	0.059035000
6	-1.031397000	-0.106603000	-0.015777000
6	-2.297589000	0.473808000	0.009107000
6	-2.449610000	1.854173000	0.066084000
6	-1.335857000	2.681916000	0.093102000
1	0.762979000	-1.540935000	-0.821155000
1	-3.175651000	-0.155476000	-0.016213000
1	-1.451814000	3.756079000	0.128366000
1	-3.443713000	2.279938000	0.085938000
7	-0.842264000	-1.537277000	-0.087059000
6	-0.774870000	-2.169029000	1.247314000
1	-0.493624000	-3.214652000	1.126758000
1	-0.018641000	-1.674966000	1.849961000

1	-1.742668000	-2.121296000	1.759750000
6	-1.792936000	-2.235667000	-0.959952000
1	-2.803489000	-2.288419000	-0.539460000
1	-1.838671000	-1.740695000	-1.927802000
1	-1.440735000	-3.256155000	-1.106865000

Conformer 2

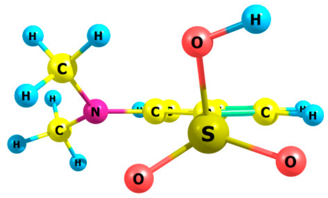

8	2.213574000	-0.510639000	1.228787000
8	2.587683000	1.076883000	-0.611969000
8	1.613124000	-1.199400000	-1.057180000
16	1.699061000	-0.010790000	-0.247745000
1	0.967242000	2.590776000	0.124432000
6	0.067451000	0.658728000	0.047891000
6	0.031782000	2.052480000	0.134044000
6	-1.132068000	-0.107247000	0.024112000
6	-2.332488000	0.636143000	0.020122000
6	-2.350624000	2.015618000	0.089866000
6	-1.166340000	2.740589000	0.167502000
1	2.819185000	0.168443000	1.557519000
1	-3.270989000	0.104435000	-0.001706000
1	-1.174217000	3.819154000	0.227277000
1	-3.303511000	2.528611000	0.100151000
7	-1.181290000	-1.484134000	-0.032149000
6	-0.375330000	-2.337590000	0.831084000
1	0.220736000	-3.041289000	0.249578000
1	0.293917000	-1.750931000	1.448627000
1	-1.034516000	-2.900498000	1.501504000
6	-2.365611000	-2.141633000	-0.548988000
1	-3.160220000	-2.256237000	0.201446000
1	-2.765432000	-1.599385000	-1.403209000
1	-2.083823000	-3.138776000	-0.887566000

Conformer 3

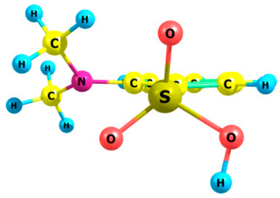

8	2.618952000	0.947406000	-0.706207000
8	1.592582000	-1.281266000	-1.007013000
8	2.246517000	-0.425238000	1.268756000
16	1.679439000	-0.204792000	-0.040939000
1	1.101308000	2.507098000	0.305980000
6	0.097055000	0.631491000	0.088811000
6	0.142231000	2.019628000	0.221052000
6	-1.141108000	-0.059177000	0.002698000
6	-2.294442000	0.751061000	-0.058132000
6	-2.233725000	2.128857000	0.032926000

6	-1.015080000	2.777256000	0.199088000
1	2.559565000	0.851634000	-1.667604000
1	-3.260005000	0.276220000	-0.138279000
1	-0.963428000	3.852200000	0.293693000
1	-3.152481000	2.699526000	-0.002343000
7	-1.253134000	-1.434982000	-0.051467000
6	-0.543383000	-2.305053000	0.879868000
1	-0.002777000	-3.089236000	0.349189000
1	0.167455000	-1.747171000	1.479881000
1	-1.259017000	-2.771395000	1.567139000
6	-2.454855000	-2.035864000	-0.594784000
1	-3.281781000	-2.082178000	0.127845000
1	-2.790676000	-1.494543000	-1.476960000
1	-2.221816000	-3.057020000	-0.896710000

Anion

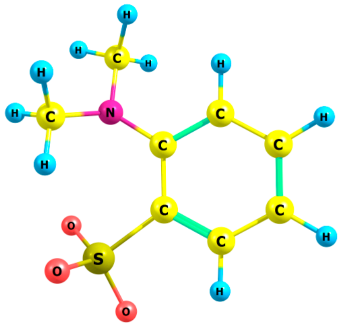

8	2.121387000	-0.524506000	1.261357000
8	2.630785000	1.100466000	-0.522740000
8	1.714500000	-1.121388000	-1.106338000
16	1.809507000	-0.048171000	-0.105025000
1	0.986104000	2.566103000	0.086986000
6	0.104106000	0.650990000	0.028956000
6	0.043749000	2.037473000	0.101232000
6	-1.104524000	-0.094535000	0.006171000
6	-2.312399000	0.629226000	0.033209000
6	-2.345196000	2.015534000	0.091756000
6	-1.160542000	2.732733000	0.136622000
1	-3.249140000	0.090788000	0.026076000
1	-1.165423000	3.814693000	0.180615000
1	-3.301899000	2.524907000	0.112002000
7	-1.147714000	-1.489891000	-0.098933000
6	-0.414271000	-2.331939000	0.832410000
1	-0.063662000	-3.230288000	0.319998000
1	0.452299000	-1.817222000	1.233521000
1	-1.064975000	-2.639864000	1.669304000
6	-2.350439000	-2.114022000	-0.585717000
1	-3.150700000	-2.203715000	0.172164000
1	-2.751621000	-1.568266000	-1.439377000
1	-2.105384000	-3.126485000	-0.914408000

2-SO2F-BSA

Conformer 1

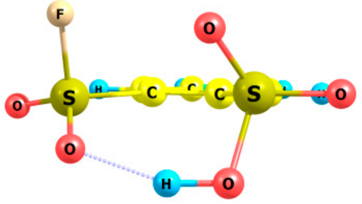

1	-2.500953000	1.860019000	-0.068803000
1	1.159132000	4.084265000	0.001121000
6	0.630143000	3.141437000	-0.004874000
6	-0.754305000	3.115560000	-0.024280000
6	-1.422899000	1.897468000	-0.037485000
6	-0.703453000	0.707724000	-0.022855000
6	0.700788000	0.727297000	0.001181000
6	1.352325000	1.952216000	0.007220000
16	1.778361000	-0.732587000	0.105201000
8	3.138671000	-0.262043000	0.129551000
8	1.255532000	-1.587186000	1.144005000
8	1.584507000	-1.424582000	-1.320644000
1	0.691519000	-1.830736000	-1.336789000
16	-1.713423000	-0.770950000	-0.112510000
8	-3.080357000	-0.409979000	-0.353508000
9	-1.648685000	-1.273119000	1.389248000
8	-1.082791000	-1.796582000	-0.910604000
1	-1.322349000	4.034918000	-0.033817000
1	2.431704000	1.958958000	0.028147000

Conformer 2

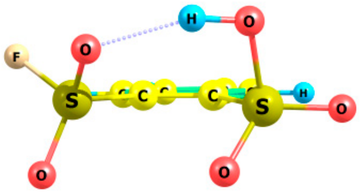

1	2.487185000	1.832920000	0.059174000
1	-1.147151000	4.083281000	-0.072911000
6	-0.631427000	3.133694000	-0.044435000
6	0.752974000	3.089563000	0.002092000
6	1.409389000	1.865894000	0.033804000
6	0.676204000	0.684206000	0.019337000
6	-0.724427000	0.723357000	-0.008148000
6	-1.366017000	1.954063000	-0.045735000
16	-1.799225000	-0.731091000	0.117401000
8	-1.254840000	-1.565341000	1.163429000
8	-3.161278000	-0.267678000	0.148159000
8	-1.616551000	-1.447832000	-1.299262000
1	-0.731447000	-1.868838000	-1.309559000
16	1.613720000	-0.851222000	0.075625000
8	2.049003000	-1.165321000	1.400957000
9	2.917981000	-0.339955000	-0.703922000
8	1.047568000	-1.847276000	-0.803495000
1	1.331783000	4.002262000	0.011625000
1	-2.445501000	1.969904000	-0.061243000

Conformer 3

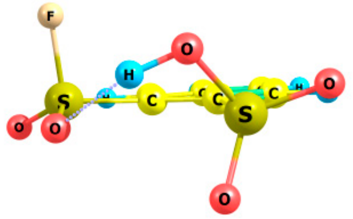

1	1.022929000	4.091901000	0.151746000
6	0.526932000	3.134045000	0.079188000
6	-0.851021000	3.065663000	-0.046707000
6	-1.472059000	1.827619000	-0.127321000
6	-0.714941000	0.659253000	-0.094994000
6	0.683230000	0.719557000	0.016890000
6	1.284898000	1.969542000	0.107894000
16	1.874015000	-0.657158000	-0.098910000
8	1.798841000	-1.177119000	-1.442596000
8	3.115140000	-0.176995000	0.451515000
8	1.331449000	-1.741527000	0.939544000
1	0.591834000	-2.219514000	0.509714000
1	-1.448204000	3.965796000	-0.077709000
1	2.359289000	2.015293000	0.201178000
8	-2.983741000	-0.564550000	-0.714883000
16	-1.709489000	-0.831904000	-0.116607000
9	-1.972161000	-0.959329000	1.459268000
8	-0.962418000	-2.015562000	-0.466111000
1	-2.545216000	1.757418000	-0.219508000

Anion

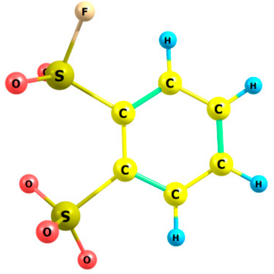

1	-2.422726000	1.879273000	-0.055380000
1	1.274192000	4.023799000	0.029990000
6	0.729639000	3.087719000	0.021209000
6	-0.659717000	3.087026000	-0.013559000
6	-1.345967000	1.882978000	-0.027948000
6	-0.640684000	0.677348000	0.000680000
6	0.755820000	0.661025000	0.016163000
6	1.420537000	1.886889000	0.030453000
16	1.821111000	-0.847406000	-0.005126000
8	1.038178000	-1.791036000	-0.814156000
8	3.057272000	-0.370826000	-0.643816000
8	1.963130000	-1.187403000	1.414176000
16	-1.624178000	-0.844583000	-0.045668000
8	-1.921334000	-1.254189000	-1.391103000
9	-3.041235000	-0.164957000	0.452252000
8	-1.321790000	-1.761549000	1.012476000
1	-1.213522000	4.016530000	-0.033968000
1	2.500825000	1.858946000	0.019192000

2-OCH3-BSA

Conformer 1

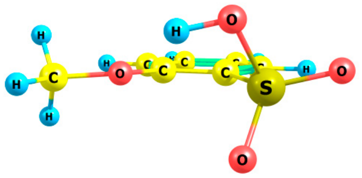

8	1.866750000	0.849639000	-1.160272000
8	2.460847000	-1.315235000	-0.215568000
8	1.913216000	0.602423000	1.315172000
16	1.679866000	-0.146099000	0.100583000
1	0.375344000	-2.652402000	-0.002196000
6	-0.055858000	-0.569715000	0.001178000
6	-0.409110000	-1.909809000	-0.002592000
6	-1.037879000	0.432670000	-0.016859000
6	-2.379067000	0.060418000	-0.018902000
6	-2.723993000	-1.288050000	-0.009235000
6	-1.750164000	-2.275208000	-0.005517000
1	1.379624000	1.663855000	-0.952376000
1	-3.155521000	0.809485000	-0.032943000
1	-2.026079000	-3.319817000	-0.004983000
1	-3.770796000	-1.560525000	-0.010544000
8	-0.602073000	1.721401000	-0.054918000
6	-1.553534000	2.776131000	0.059866000
1	-0.975831000	3.695384000	0.081198000
1	-2.231955000	2.791533000	-0.795225000
1	-2.124934000	2.685611000	0.984801000

Conformer 2

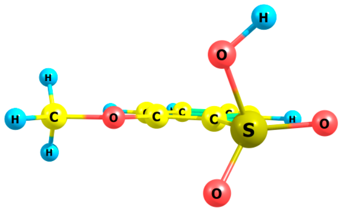

8	-1.898814000	0.789693000	1.225740000
8	-2.467431000	-1.285192000	0.010855000
8	-1.879159000	0.825320000	-1.229327000
16	-1.672058000	-0.079758000	-0.130594000
1	-0.447258000	-2.611148000	-0.040396000
6	0.050767000	-0.542310000	-0.025854000
6	0.360801000	-1.895060000	-0.019714000
6	1.065741000	0.431979000	-0.000993000
6	2.394144000	0.005420000	0.027045000
6	2.693035000	-1.352478000	0.028589000
6	1.686978000	-2.307545000	0.006556000
1	-2.292981000	0.194655000	1.879523000
1	3.195289000	0.727740000	0.047791000
1	1.926333000	-3.361075000	0.007696000
1	3.730042000	-1.660268000	0.048537000
8	0.678626000	1.720628000	0.006692000
6	1.662853000	2.745835000	-0.005345000
1	1.110396000	3.680294000	-0.019972000
1	2.288473000	2.706498000	0.889261000
1	2.290944000	2.680799000	-0.896313000

Conformer 3

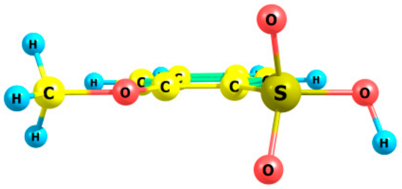

8	-2.522906000	-1.212249000	-0.015607000
8	-1.904152000	0.855003000	-1.208058000
8	-1.880794000	0.760008000	1.309800000
16	-1.630131000	0.152537000	0.029025000
1	-0.568135000	-2.564618000	0.043335000
6	0.032336000	-0.517256000	0.004412000
6	0.268042000	-1.883825000	0.018889000
6	1.102843000	0.395708000	-0.008498000
6	2.405663000	-0.100552000	-0.019306000
6	2.630047000	-1.472795000	-0.009798000
6	1.571462000	-2.367781000	0.010893000
1	-2.897598000	-1.268002000	-0.906298000
1	3.244344000	0.577908000	-0.032845000
1	1.750297000	-3.433246000	0.023363000
1	3.648161000	-1.838293000	-0.016737000
8	0.780595000	1.703718000	-0.009547000
6	1.813404000	2.679868000	-0.017931000
1	1.307352000	3.640297000	-0.014198000
1	2.443510000	2.596845000	0.870255000
1	2.429441000	2.596481000	-0.915949000

Anion

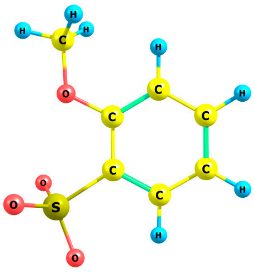

8	-1.907129000	0.807371000	1.240394000
8	-2.506988000	-1.244535000	-0.000258000
8	-1.907256000	0.807943000	-1.239879000
16	-1.763090000	0.029878000	-0.000005000
1	-0.566175000	-2.540696000	-0.000104000
6	-0.002810000	-0.510367000	-0.000071000
6	0.279257000	-1.866797000	-0.000083000
6	1.068578000	0.400675000	-0.000115000
6	2.383370000	-0.069675000	-0.000024000
6	2.639640000	-1.439973000	0.000093000
6	1.590379000	-2.343409000	-0.000004000
1	3.212969000	0.622208000	-0.000202000
1	1.781881000	-3.409331000	0.000088000
1	3.666097000	-1.787325000	0.000164000
8	0.754992000	1.723265000	-0.000279000
6	1.790493000	2.672608000	0.000135000
1	1.303276000	3.645292000	0.000378000
1	2.424398000	2.590273000	0.890664000
1	2.424592000	2.590808000	-0.890323000

2-COOH-BSA

Conformer 1

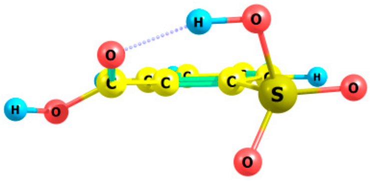

8	2.016088000	0.539829000	-1.177957000
8	2.452015000	-1.485665000	0.089307000
8	1.809647000	0.616842000	1.305341000
16	1.693764000	-0.261447000	0.162137000
6	-0.060769000	-0.673269000	-0.023349000
6	-1.088525000	0.286785000	-0.020469000
6	-2.412742000	-0.155121000	-0.033765000
6	-2.713973000	-1.509832000	-0.072017000
6	-1.690833000	-2.444934000	-0.086118000
6	-0.365083000	-2.025209000	-0.053257000
1	1.463965000	1.359800000	-1.145421000
1	-3.207106000	0.575592000	-0.025885000
1	-3.746840000	-1.828563000	-0.090194000
1	-1.915839000	-3.502175000	-0.113995000
1	0.444974000	-2.738876000	-0.039744000
6	-0.839878000	1.753779000	-0.081515000
8	0.103702000	2.298834000	-0.619225000
8	-1.820483000	2.470006000	0.487011000
1	-1.596296000	3.405410000	0.368162000

Conformer 2

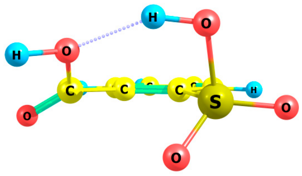

8	-2.075274000	0.145351000	1.245309000
8	-2.240129000	-1.722956000	-0.292939000
8	-1.830301000	0.589164000	-1.196584000
16	-1.621156000	-0.426493000	-0.188491000
6	0.158417000	-0.654539000	0.013786000
6	1.077728000	0.406673000	0.007017000
6	2.439442000	0.105148000	0.030503000
6	2.880889000	-1.208950000	0.095034000
6	1.961392000	-2.247035000	0.117314000
6	0.600517000	-1.969319000	0.064426000
1	-1.698751000	1.041712000	1.323433000
1	3.145571000	0.922177000	-0.001226000
1	3.941443000	-1.417176000	0.121522000
1	2.295722000	-3.274299000	0.160834000
1	-0.128690000	-2.765059000	0.043182000
6	0.753101000	1.864999000	-0.034891000
8	1.393568000	2.681260000	-0.637069000
8	-0.299044000	2.226979000	0.755483000
1	-0.436272000	3.176289000	0.615376000

Conformer 3

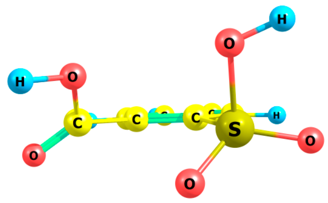

8	-2.122466000	0.131907000	1.260046000
8	-2.268346000	-1.593342000	-0.486605000
8	-1.788471000	0.802903000	-1.092881000
16	-1.620418000	-0.324565000	-0.214297000
6	0.140555000	-0.635389000	0.014547000
6	1.107936000	0.379313000	0.018800000
6	2.451667000	0.010942000	0.043312000
6	2.829141000	-1.323491000	0.100709000
6	1.861189000	-2.317130000	0.110129000
6	0.516642000	-1.973135000	0.051276000
1	-2.607375000	-0.617898000	1.634534000
1	3.196124000	0.793587000	0.016209000
1	3.878350000	-1.583374000	0.128537000
1	2.145320000	-3.359762000	0.142809000
1	-0.246740000	-2.734766000	0.005215000
6	0.839350000	1.854152000	-0.010421000
8	1.472766000	2.628636000	-0.678971000
8	-0.124335000	2.223638000	0.848724000
1	-0.271066000	3.173739000	0.728832000

Anion

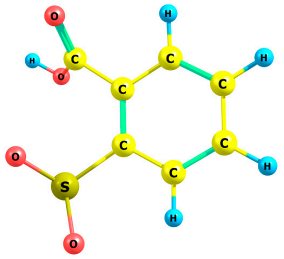

8	2.158908000	-1.626483000	-0.709460000
8	2.126411000	-0.209678000	1.314028000
8	1.826367000	0.815799000	-0.927156000
16	1.689037000	-0.380793000	-0.078596000
6	-0.133458000	-0.631392000	0.036816000
6	-1.065540000	0.412385000	0.014093000
6	-2.432435000	0.123778000	0.004526000
6	-2.882679000	-1.186417000	0.064562000
6	-1.957309000	-2.224092000	0.104719000
6	-0.597944000	-1.943746000	0.075706000
1	-3.135717000	0.944290000	-0.052313000
1	-3.945162000	-1.395248000	0.067344000
1	-2.294975000	-3.253049000	0.139314000
1	0.136735000	-2.736058000	0.051377000
6	-0.699285000	1.862328000	-0.032960000
8	-1.142264000	2.648692000	-0.838296000
8	0.086613000	2.236312000	0.991677000
1	0.378160000	3.138546000	0.800691000
